# Genome-wide analysis of the rice PPR gene family and their expression profiles under different stress treatments

**DOI:** 10.1186/s12864-018-5088-9

**Published:** 2018-10-01

**Authors:** Guanglong Chen, Yu Zou, Jihong Hu, Yi Ding

**Affiliations:** 1 State Key Laboratory of Hybrid Rice, Department of Genetics, College of Life Sciences, Wuhan University, Wuhan, 430072 China; 2 Key Laboratory of Biology and Genetic Improvement of Oil Crops, Ministry of Agriculture, Oil Crops Research Institute of the Chinese Academy of Agricultural Sciences, Wuhan, 430062 China

**Keywords:** Rice (*Oryza sativa* L.), Pentatricopeptide-repeat protein, miRNA, Expression patterns, Salinity or drought stress

## Abstract

**Background:**

Pentatricopeptide-repeat proteins (PPRs) are characterized by tandem arrays of a degenerate 35-amino-acid (PPR motifs), which can bind RNA strands and participate in post-transcription. PPR proteins family is one of the largest families in land plants and play important roles in organelle RNA metabolism and plant development. However, the functions of *PPR* genes involved in biotic and abiotic stresses of rice (*Oryza sativa* L.) remain largely unknown.

**Results:**

In the present study, a comprehensive genome-wide analysis of *PPR* genes was performed. A total of 491 *PPR* genes were found in the rice genome, of which 246 *PPR* genes belong to the P subfamily, and 245 genes belong to the PLS subfamily. Gene structure analysis showed that most *PPR* genes lack intron. Chromosomal location analysis indicated that *PPR* genes were widely distributed in all 12 rice chromosomes. Phylogenetic relationship analysis revealed the distinct difference between the P and PLS subfamilies. Many PPR proteins are predicted to target chloroplasts or mitochondria, and a PPR protein (LOC_Os10g34310) was verified to localize in mitochondria. Furthermore, three *PPR* genes (*LOC_Os03g17634*,*LOC_Os07g40820*,*LOC_Os04g51350*) were verified as corresponding miRNA targets. The expression pattern analysis showed that many *PPR* genes could be induced under biotic and abiotic stresses. Finally, seven *PPR* genes were confirmed with their expression patterns under salinity or drought stress.

**Conclusions:**

We found 491 *PPR* genes in the rice genome, and our genes structure analysis and syntenic analysis indicated that *PPR* genes might be derived from amplification by retro-transposition. The expression pattern present here suggested that PPR proteins have crucial roles in response to different abiotic stresses in rice. Taken together, our study provides a comprehensive analysis of the *PPR* gene family and will facilitate further studies on their roles in rice growth and development.

**Electronic supplementary material:**

The online version of this article (10.1186/s12864-018-5088-9) contains supplementary material, which is available to authorized users.

## Background

Pentatricopeptide repeat (PPR) proteins are a group of proteins that contain tandem repeats of degenerate 35-amino-acid motifs (PPR motifs) [1, 2], which play an important role in plant growth and development. Since the first PPR protein was recognized and characterized in the *Arabidopsis thaliana* genome sequence [3], many PPR proteins have been identified in plants. As RNA-binding proteins, PPR proteins participate in posttranscriptional processes including RNA editing [4–6], splicing [7], stability [8], cleavage [9], degradation [10], and translation [11]. A study has shown that the structure of the canonical PPR motif (P motif) is a hairpin of two α-helices [12]. A PPR motif can recognize a single RNA nucleotide [12, 13], and an array of PPR domains can recognize special single-stranded RNA targets. In contrast to CRISPR gene editing at the DNA level, PPR–RNA interactions may become another editing alternative at the post-transcriptional level [1, 14].

Variants of the P motif include the L (long) motif and S (small) motif. Based on the P, L and S motifs, PPR proteins can be divided into two subfamilies, P and PLS [4]. The PLS subfamily usually has an E or DYW domain in the C-terminal region [1]. Furthermore, the PLS subfamily can also be divided into three subgroups, including the PLS subgroup, E subgroup and DYW subgroup. Recently, a study on the structural motifs had redefined 10 different variants of the P motif in plant proteins, including one canonical P motif and nine P-like motifs (P1, P2, L1, L2, S1, S2, SS, E1 and E2) [15]. Therefore, P subfamily proteins contain tandem arrays of P motifs without other PPR motifs, while PLS subgroup proteins only contain varied PPR motifs, sometimes also with a non-PPR motif, DYW.

The number of PPR proteins is relatively large in many land plants compared with other eukaryotic organisms. For example, there are 441 *PPR* genes in *Arabidopsis thaliana* [4], and 471 *PPR* genes in tomato [16]. Surprisingly, the lycophyte *Selaginella moellendorffii* has 1670 *PPR* genes [17]. However, only 6, 2 and 2 PPR proteins were found in *Homo sapiens*, *Caenorhabditis elegans* and *Drosophila*, respectively [2, 4].

Studies have shown that most PPR proteins are targeted to mitochondria or chloroplasts and the function of some PPR proteins was elucidated to be associated with chloroplast, seed development, and fertility [1, 2]. It was reported that the nuclear *OGR1* gene encoded a DYW- PPR protein, which is essential for mitochondria, and the of *ogr1* mutant exhibited delayed seed germination and sterility [18]. The well-known function of some PPR proteins is acting as *Rf* (restorer of fertility) genes associated with cytoplasmic male sterility (CMS) of plants. Many *PPR* genes play the roles of restoring fertility in land plants, such as *RFL2* in *Arabidopsis* [13], *Rf1* in sorghum (*Sorghum bicolor* L.) [19], *CaPPR6* in pepper (*Capsicum annuum*l.) [20] and *RF592* in petunia [21]. Previous studies revealed that CMS is caused by the accumulation of the ORF79 protein and can be restored by two PPR proteins, Rf1a and Rf1b, in BT-type cytoplasmic male sterile rice (*Oryza sativa* L.), [22, 23]. However, most Rf-related *PPR* genes belong to the P subfamily, which lacks the E/DYW motif [24].

PPR proteins play diverse and important roles in plant developmental processes and responses to biotic and abiotic stresses. For instance, SOAR1, an *Arabidopsis* cytosol-nucleus dual-localized PPR protein, was reported to be involved in ABA signaling and tolerance to drought, salinity or cold stress [25]. In addition, in *Arabidopsis,* five mitochondrial PPR proteins, PPR40 [26], ABO5 [27], SLG1 [28], PGN [29], and SLO2 [30], were reported to be involved in abiotic stress, including salt or drought stress responses. In rice, *OsV4* encoding a PPR protein is required for chloroplast development in early stages under cold stress [31]. A novel thermosensitive chlorophyll-deficient mutant, *tcd10*, encoding a PPR protein (LOC_Os10g28600) was also reported to be required for chloroplast development and photosynthesis in rice under cold stress [32]. However, few studies on *PPR* genes of rice involved in salinity and drought stress have been reported.

In the present study, we characterized the *PPR* genes in the rice genome with the report of redefining the structural of PPR motifs and analyzed the chromosomal arrangement, genes structure and the consensus sequence of PPR motifs as well as the subcellular localization. The expression patterns of the *PPR* genes in response to biotic and abiotic stresses were also analyzed, especially, under salt and drought stresses. Furthermore, the expression patterns were examined by quantitative real-time RT-PCR. These results will provide a biological reference for further elucidating the role of *PPR* genes in rice.

## Results

### Identification of *PPR* genes in the rice genome

**Fig. 1:**
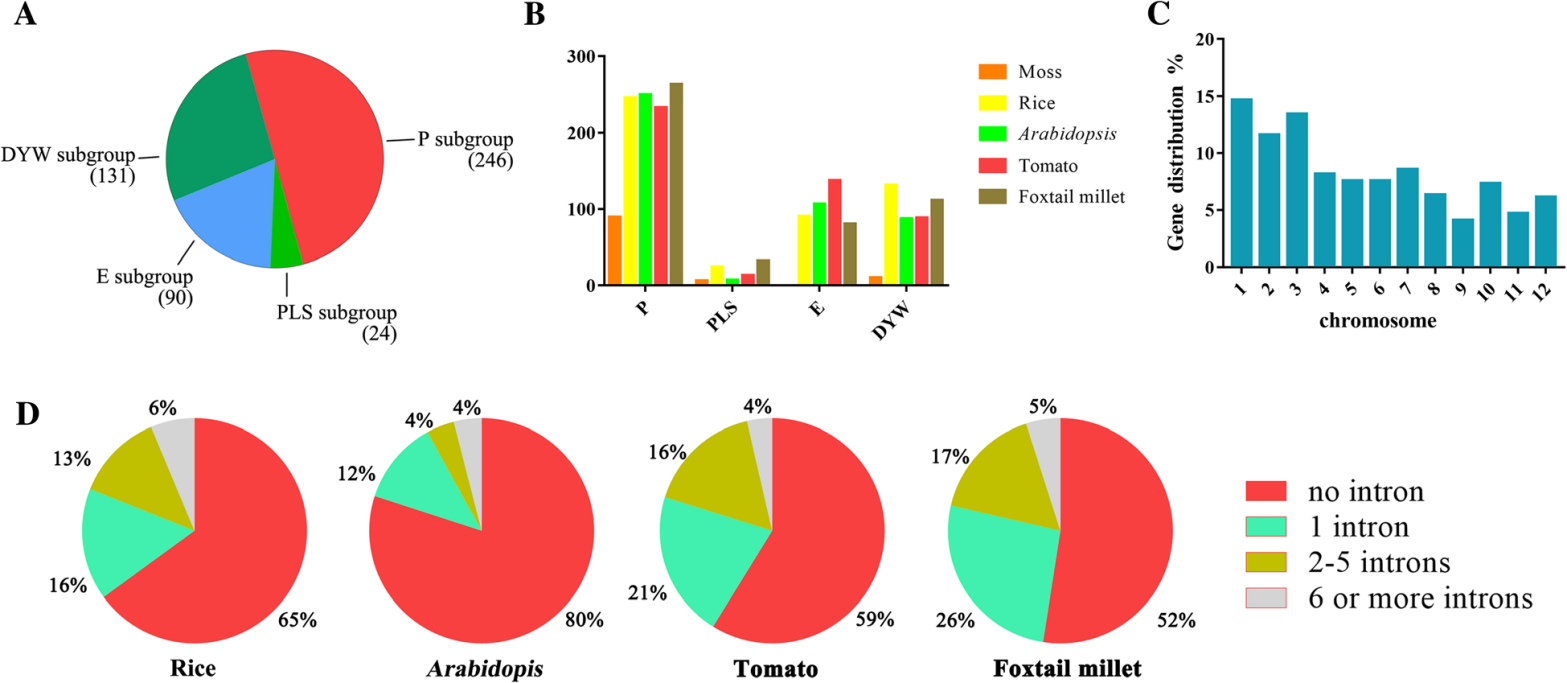
Numbers of *PPR* genes and their intron numbers in rice and other species**. a** PPR proteins in a rice:P subgroup (246), PLS subgroup (24), E subgroup (90) and DYW subgroup (131). **b** Numbers and subclasses of *PPR* genes in moss, rice, Arabidopsis, tomato and foxtail millet,. **c** *PPR* genes distribution on rice 12 chromosomes. Chromosome 1 contained the most *PPR* genes (14.6%) and chromosome 9 had the fewest *PPR* genes (4%). **d** Number of introns in rice, *Arabidopsis*, tomato and foxtail millet

A total of 491 *PPR* genes were identified in the rice genome in this study (Additional file 1: Table S1). First, we identified 477 *PPR* genes in the RGAP and RAP-DB database at the beginning of the analysis. This number corresponded with that of previous reports [33]. Second, 14 *PPR* genes were found by comparing our result with those of Cheng et al. [15]. Combined the two results, there should be a total of 491 *PPR* genes in the rice genome. The 491 PPRs contained 246 P-class and 245 PLS-class members based on the proteins structures. Furthermore, there were 90 E and 131 DYW sub-groups in the PLS subfamily, respectively (Fig. 1a and Additional file 1: Table S1). The numbers of *PPR* genes in rice genome were higher than the 105 in moss (89 P-type and 16 PLS-type) [34], and 450 in *Arabidopsis thaliana* (251 P-type and 199 PLS-type) [33], and were relatively close to the 471 in tomato (233 P-type and 238 PLS-type) [16] and 486 in foxtail millet (263 P-type and 223 PLS-type) [17] (Fig. 1b and Table 1).

**Table 1:** The number of PPR proteins in rice and other species

Plant species	Total	P	PLS	E	DYW
Moss	105	89	6	0	10
Rice	491	246	24	90	131
*Arabidopsis*	441	241	6	107	87
Tomato	471	233	13	137	88
Foxtail millet	486	263	32	111	80

### Structure analysis of PPR proteins

The arrangement of PPR motifs in rice obeyed the rules that the P class contained a series of canonical P motifs and the PLS-class had tandem repeat P-like motifs (Additional file 2: Figure S1A). Most PLS subfamily proteins were arranged in the manner of P1-L1-S1, or P2-L2-S2 and an E or DYW domain in the C-terminal region. In our study, only 9 PPR proteins (LOC_Os01g55290, LOC_Os01g62400, LOC_Os03g18620, LOC_Os04g21470, LOC_Os06g44820, LOC_Os07g36450, LOC_Os10g35650, LOC_Os11g03200, LOC_Os12g02950) contained two P motifs, while LOC_Os04g43430 had 28 motifs, including 27 P-like motifs and one DYW motif.

To obtain consensus sequences of motifs, we performed WebLogo [35] with default parameters using all the sequences of each PPR motif. Most P1 and P2 motifs started with two valines (Val, V) and ended with proline (Pro, P) and aspartic acid (Asp, D) (Additional file 2: Figure S1B). P1 and P2 were very similar. Glycine (Gly, G) was in all motif sequences at 15th position, while most motifs possessed glutamic acid (Glu, E) in 18th and 19th position. Alanine (Ala, A) was shown at the 20th position in most PPR motif. Each motif contained methionine (Met, M) in the 27th position except L1 with V and E1 with leucine (Leu, L). From the 1 st to the 12th in the P1 motif, there were 12 amino acid residues that were same as in the P2 and SS motifs. The consensus sequence is similar to that obtained by using PPR motif sequences from 41 representative genomes [15].

### Chromosomal localization and gene structure analysis

Our study showed the 491 *PPR* genes were widely and unevenly distributed on all 12 rice chromosomes (Fig. 1c and Additional file 2: Figure S2). There were 72 *PPR* genes on chromosome 1, the largest chromosome of the rice genome. The lowest number of *PPR*s was found on chromosome 9, which only possessed 20 *PPR* genes. Sixty-six *PPR* genes mapped on chromosome 3, and fifty-seven genes in chromosome 2. We analyzed the sequence of *PPR* genes and their coding sequences, and found that the majority of *PPR* genes were intron-less (Fig. 1d and Additional file 2: Figure S3). More than 65% (319/491) of rice *PPR* gene ORFs (open reading frame) contained a single exon, 16% (79/491) had one intron, and only 19% genes had more than one intron (Fig. 1d). The result was similar to the structure of *PPR* genes in *Arabidopsis*, in which approximately 80% *PPR* genes had no intron and 12% had only one intron. Approximately 58% and 52% *PPR* genes contained only one exon in tomato and foxtail millet, respectively. Most *PPR* genes were intron-less in all *PPR* gene families. It had been presumed that the *PPR* genes family was derived from amplification by transposition [4, 33]. Some intron-rich *PPR* genes would represent “ancient” *PPR* genes and were duplicated by reverse transcription and integrated into the genome, which might create novel genes.

### Phylogenetic and syntenic analysis of rice *PPR* proteins

**Fig. 2:**
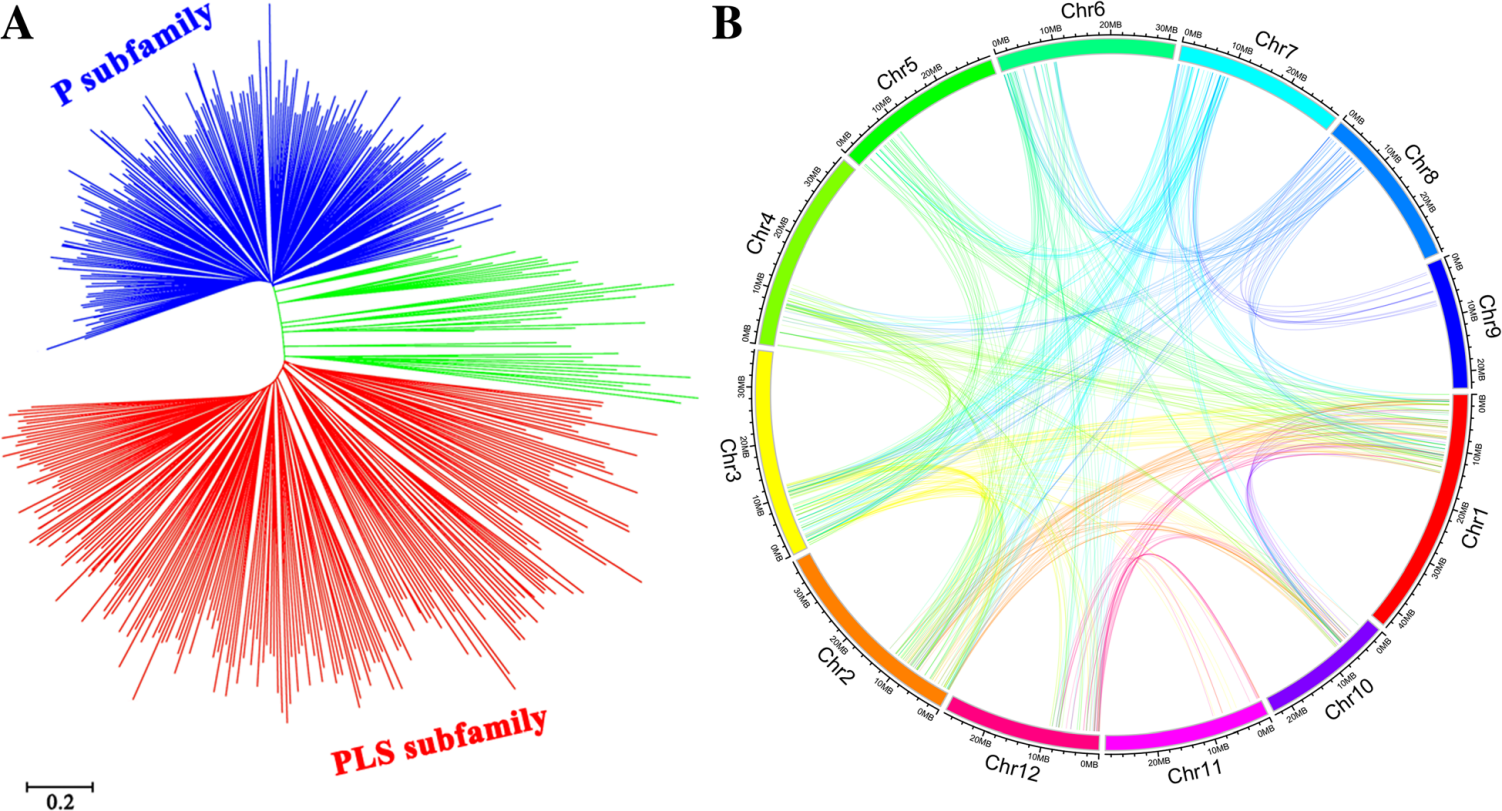
Phylogenetic analysis and synteny analysis of the *PPR* genes in rice. **a** Phylogenetic tree of 491 *PPR* genes in rice. The blue represented the P subfamily and the red represented the PLS subfamily, while the green represented the mixture of PLS proteins and P subfamily genes. Multiple sequence alignment of full-length proteins was performed by ClustalX. The phylogenetic tree was constructed using MEGA7.0 with the neighbor-joining method and 1000 bootstrap replicates. Scale bar represents 0.2 amino acid substitutions per site. **b** Synteny analysis of PPR genes in rice. Chromosome 1 to 12 are shown with different colors and in a circular form. The different color curves represent the synteny relationship of *PPR* genes in the rice genome

Using the neighbor-joining (NJ) method based on the full-length amino acid sequences of 491 rice PPR proteins, the phylogenetic tree was constructed. The tree was classified into two distinct subfamilies (P and PLS subfamily) (Fig. 2a). However, several PPR members of the PLS subfamily were clustered with the P subfamily, which is consistent with the results from the poplar phylogenetic analysis in which some PPR proteins possessed the PLS structure, but were clustered into the P subfamily [36].

To analyze the evolutionary relationships among PLS subgroup genes, we performed multiple sequence alignment of 24 from rice, 6 form *Arabidopsis*, 6 from moss, 13 from tomato and 32 from foxtail millet (Table 1) to generate a phylogenetic tree (Additional file 1: Figure S4). Most of these PPRs were clustered with a group in the same species and the moss PPRs were obviously divergent from other plants. However, some PPRs from rice, *Arabidopsis* and tomato were mixed, suggesting that these PPR members were homologous. Furthermore, we evaluated the syntenic relations of *PPR* genes using MCScanX program. A total of 276 *PPR* genes were located within syntenic blocks on all rice chromosomes (Fig. 2b).

### Subcellular localization of PPR proteins

**Fig. 3:**
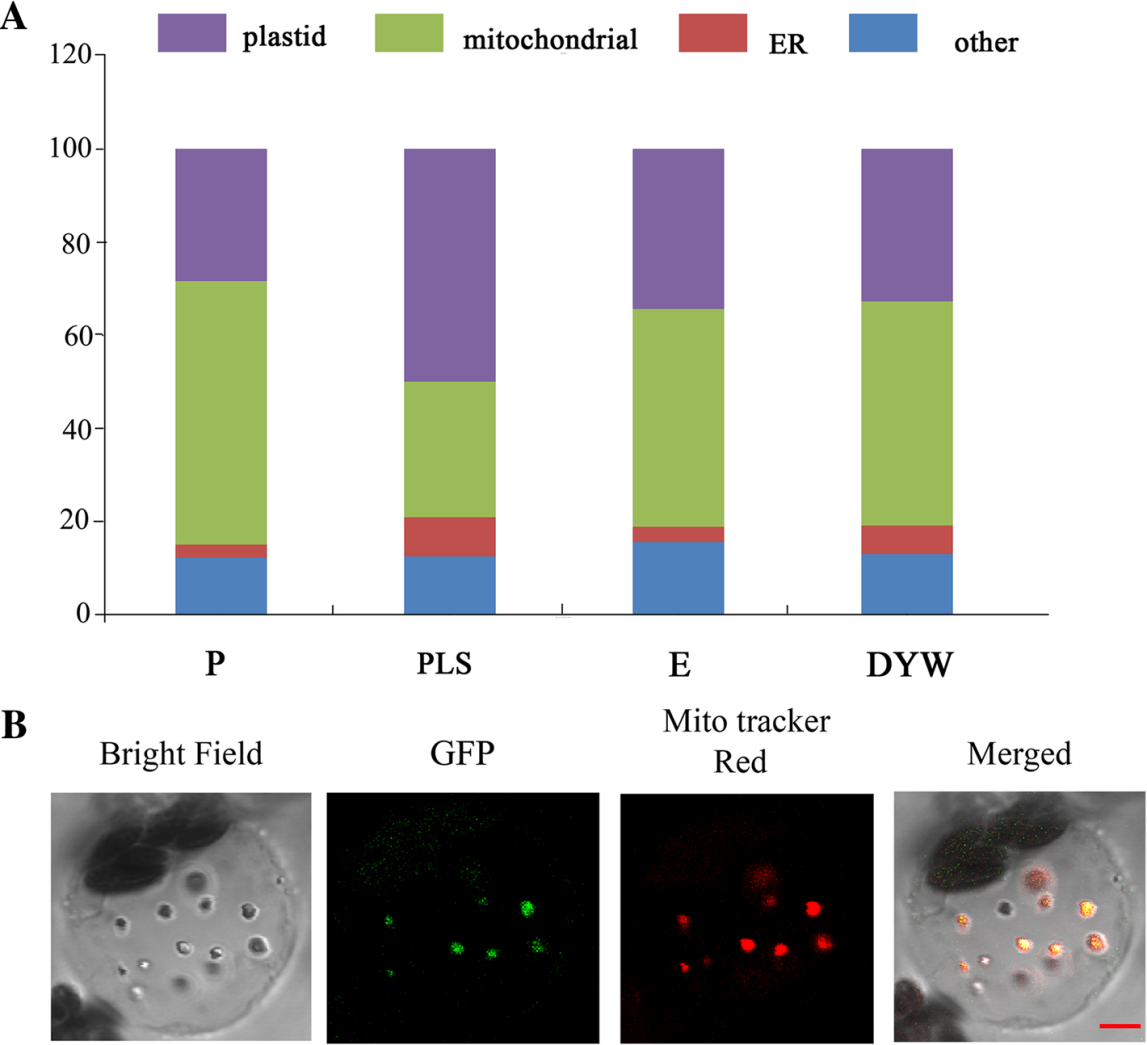
Subcellular localization of a *PPR* gene in rice. **a** The results of subcellular localization of each subgroup and species using TargetP and Predotar on-line tools. Plastid in purple, mitochondria in green, endoplasmic reticulum (ER) in red, and other locations in blue. **b** Confocal scanning microscopy showing the subcellular localization of a PPR (*LOC_Os10g34310*) protein in mitochondria. Cells were treated with MitoTracker Red to detect mitochondria. GFP signals of fusion proteins localized in the chloroplasts of rice protoplasts. Bar = 5 μm

Most reported *PPR* genes play roles in mitochondria or plastids. Therefore, we predicted their subcellular localization using TargetP 1.1 [37] and Predotarv.1.04 [38] in the study. We obtained similar results with the two programs, which predicted that most PPR proteins targeted either mitochondria or plastids. Using TargetP, approximately 54% PPR proteins were predicted to target mitochondria and 28% to chloroplast, while Predotar predicted 44% to mitochondria and 22% to plastid (Additional file 1: Table S2). Combining the two results, we found that more than one-half of the P-class proteins were located in the mitochondria and approximately 28% were in plastids (Fig. 3a). For PLS-class proteins, approximately 50% were predicted in plastids and 30% in mitochondria. E-class and DYW-class proteins had a similar distribution in subcellular localization, with half in mitochondria and 30% in plastids (Fig. 3a). Furthermore, we used PPR-GFP fusion proteins to detect their subcellular localization in rice protoplasts. The protein encoded by the gene *LOC_Os10g34310* was indeed located in mitochondria (Fig. 3b). The PPR-GFP fusion protein was observed with green fluorescence in mitochondria that were treated with MitoTracker Red and could be detected with red fluorescence by a confocal laser scanning microscope. As a result, the *PPR* gene (*LOC_Os10g34310*) was verified to localize in mitochondria, which was consistent with the result predicted by TargetP (Fig. 3b).

### PPR proteins as the target genes of miRNAs in rice

**Fig. 4:**
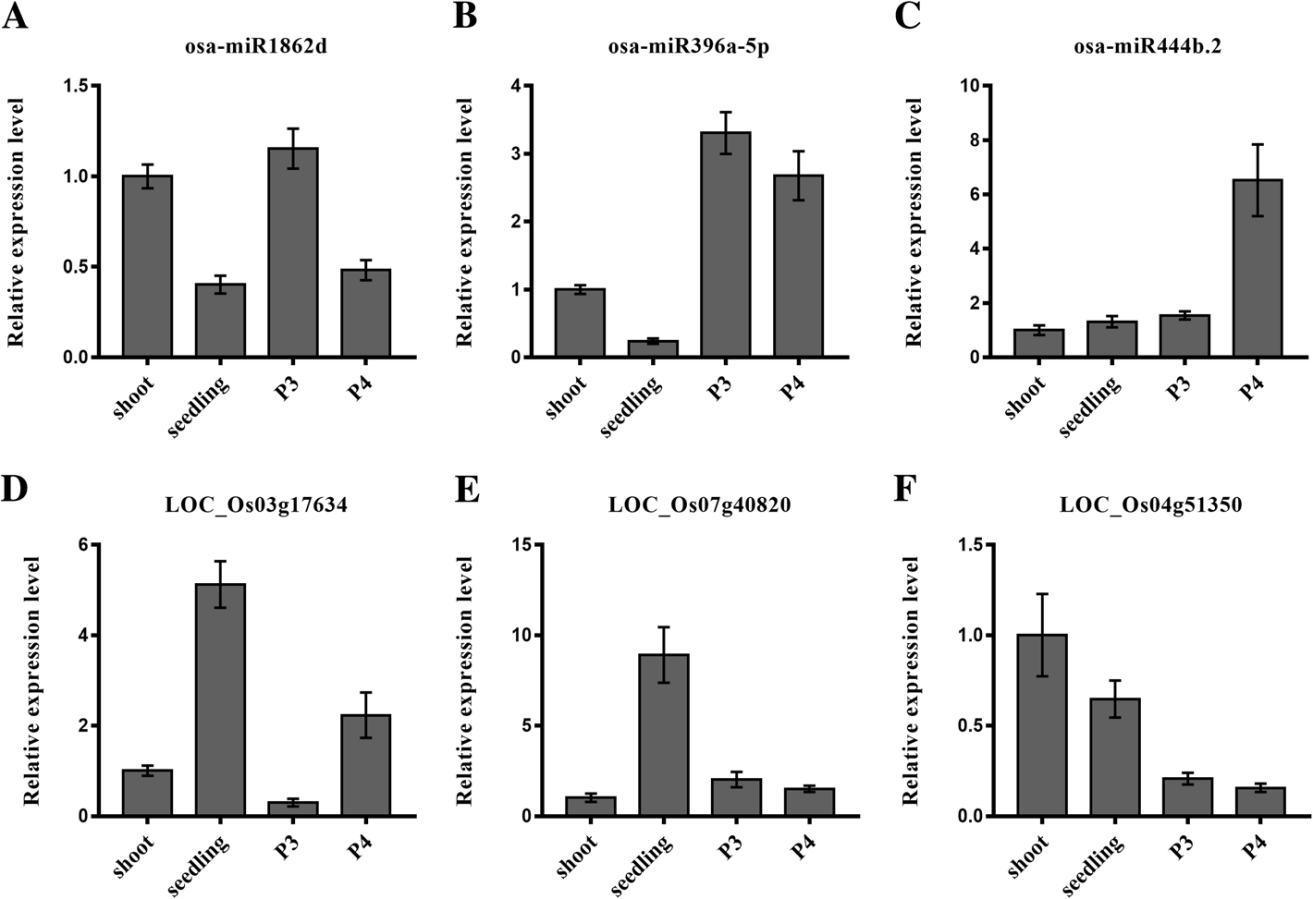
Validation and expression of selected miRNAs and their target PPR genes. Relative expression levels of three *PPR* genes as the targets of miRNAs in rice shoot, seeding, P3 and P4, **(a, d)** osa-miR1862d (**a**) and its target (*LOC_Os03g17634*)(**d**). **(b, e)** osa-miR396a-5p (**b**) and its target (*LOC_Os07g40820*) (**e**). **(c, f)** osa-miR444b.2 and its target (*LOC_Os04g51350*)

PPR proteins could be the target genes of some miRNAs, which play diverse roles in plant development, including fertility transition [39] and abiotic stress [40]. In the present study, we identified three miRNAs and their corresponding targets (*PPR* genes) and validated the expression patterns using qRT-PCR (Fig. 4). The results showed that osa-miR1862d had a higher expression in shoot and P3 stages, while *LOC_Os03g17634* was expressed in shoot and P3 stages with lower expression (Fig. 4a, and d); osa-miR396a-5p was mainly expressed in panicles, and *LOC_Os07g40820* was predominantly expressed in seedling (Fig. 4b, and e). The expression level of osa-miR444b.2 was highest in the P4 stage, but *LOC_Os04g51350* was mainly expressed in leaves (Fig. 4c, and f). These results future showed negative correlations between expression levels of miRNAs and their corresponding PPR target genes at the relative expression level.

In this study, a total of 49 miRNAs were predicted to target 54 *PPR* genes (Additional file 1: Table S3). Compared with previous studies, we found that some miRNAs targeted *PPR* genes were involved in rice male sterility, and some miRNAs had differential expression in the rice male sterile lines Meixiang A and Wuxiang S [39, 41], indicating their roles in the regulation of pollen abortion and participation in rice male sterility.

### Expression patterns of *PPR* genes

**Fig. 5:**
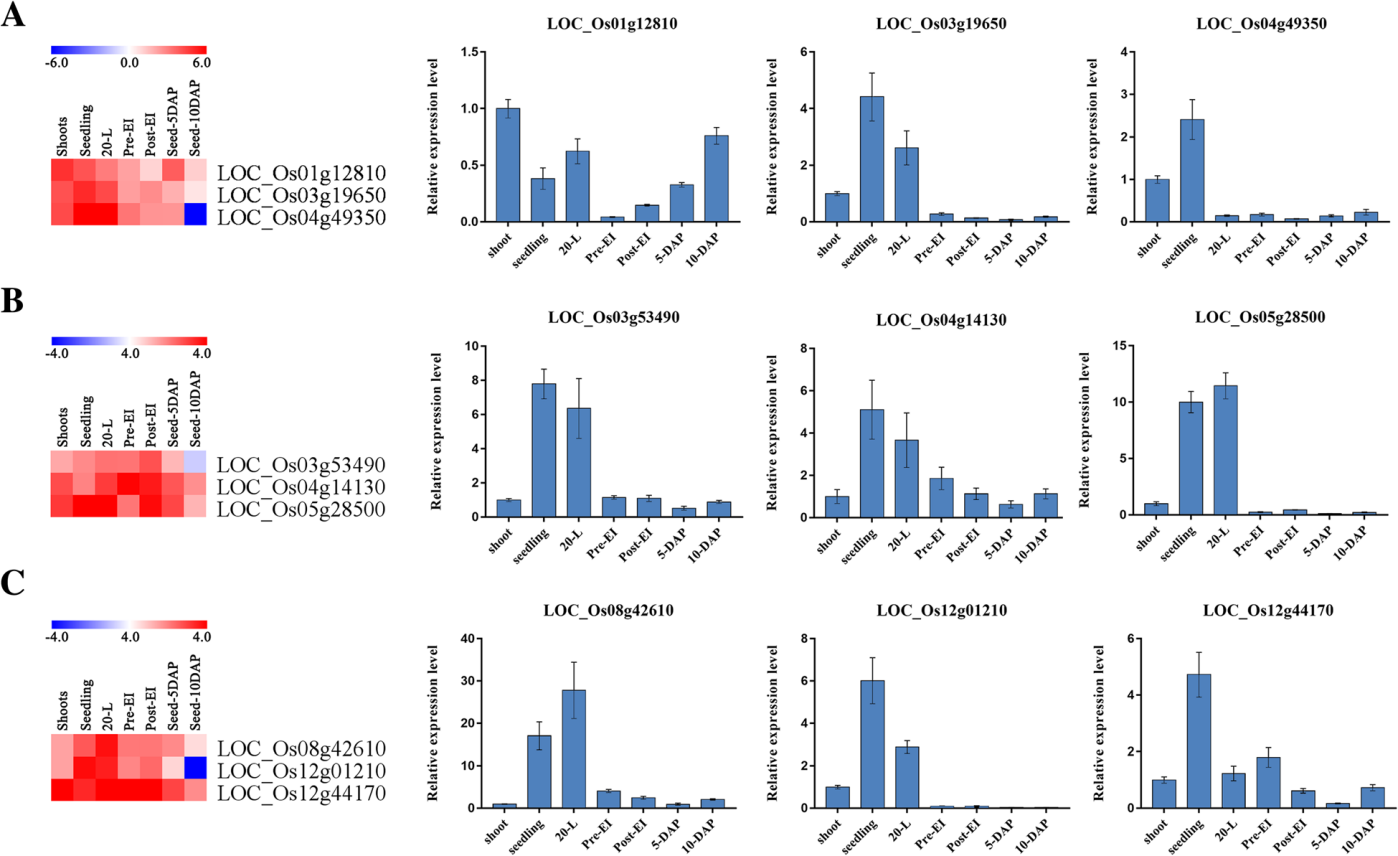
qRT-PCT validation of nine PPR gene expression patterns. **a** Heatmap and relative expression levels of *LOC_Os01g12810*, *LOC_Os03g19650* and *LOC_Os04g49350*. **b** Heatmap and relative expression levels of *LOC_Os03g53490*, *LOC_Os04g14130* and *LOC_Os05g28500*. **c** Heatmap and relative expression levels of *LOC_Os08g42610*, *LOC_Os12g01210* and *LOC_Os12g44170*

Gene expression profiles could provide clues for functional studies. We used FPKM values to represent *PPR* gene expression in different tissues and organs of rice in this study. Based on hierarchical clustering, the Log2-based RNA-seq value was used to create the heat map, which represented the relative expression of 491 *PPR* genes in different developmental stages (Additional file 2: Figure S5 and Additional file 1: Table S4). Our results showed that the *PPR* genes had different expression levels in various organs and tissues. Most *PPR* genes were expressed in young leaves and panicles, showing a lower expression in endosperm after 25 days of pollination. Then, we used qRT-PCR to verify the RNA-seq data (Fig. 5) and revealed that the genes *LOC_Os03g19650* and *LOC_Os04g49350* had high expression levels in shoot and seedling, but were expressed at an extremely low level in other examined organs (Fig. 5a). The genes *LOC_Os05g28500* and *LOC_Os12g1210* were specifically expressed in seedling and 20-day-old leaves (Fig. 5b, and c). The remaining results are as follows: Genes *LOC_Os03g53490*, *LOC_08g42610,* and *LOC_12g44170* were mainly expressed in leaves and were detected in panicles and seeds with lower expression. Gene *LOC_Os01g12180* was expressed in all examined organs (except at pre-EI stage), while *LOC_Os04g14130* exhibited less and less expression as the rice grew. Those results revealed the potential functions of *PPR* genes in different rice development stages.

### Expression regulation of *PPR* genes under biotic and abiotic stresses

**Fig. 6:**
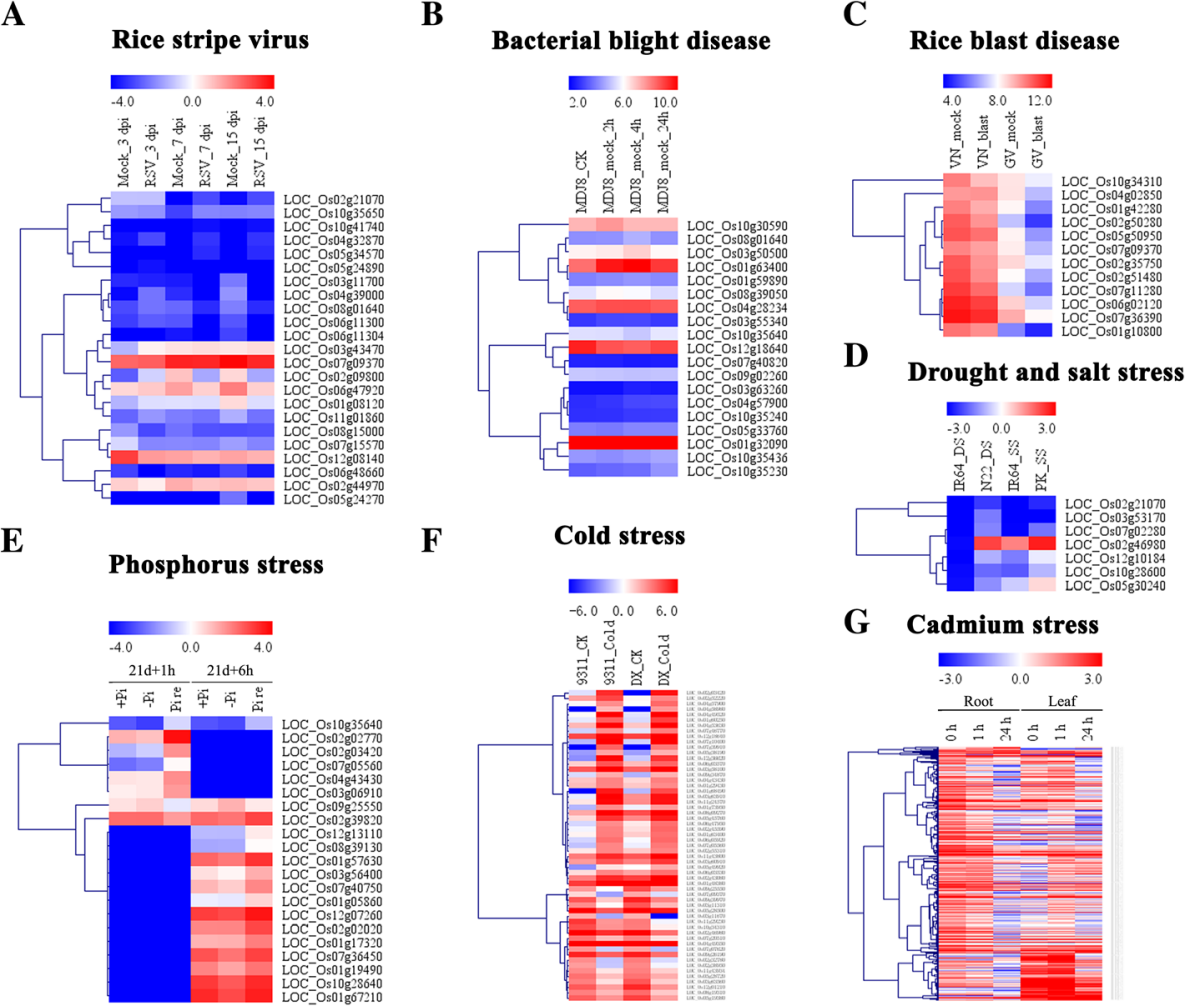
Expression profiles of the PPR genes in response to different stresses. All heat maps were generated using MeV4.9 software with log2-transformed FPKM values. **a** Expression profiles of *PPR* genes in rice infected by rice stripe virus (RSV). Mock means control and dpi means days post inoculation. **b** Expression profiles of *PPR* genes in rice infected with bacterial blight disease. MDJ8 represents *Japonica* rice cultivar Mudanjiang8. **c** Expression profile of *PPR* genes in rice infected by rice blast disease. VN and GV represent a blast-tolerant cultivar and susceptible cultivar, respectively. **d** Expression profiles of *PPR* genes in rice under drought and salt stresses, respectively. N22 is a drought-tolerant cultivar. PK is a salinity-tolerant rice cultivar and IR64 is a susceptible cultivar. **e** Expression profiles of *PPR* genes in rice under phosphorus stress. +Pi, -Pi and Pire represent control, phosphate starvation and recovery, respectively. **f** Expression profiles of *PPR* genes in rice under cold stress. 9311 and DX (Dongxiang wile rice) are two rice varieties, and CK is the control condition of cold. **g** Expression profiles of *PPR* genes in rice under cadmium stress. Rice was treated with cadmium for 1 h and 24 h, and root and shoot were collected for this experiment

Previous studies revealed that many PPR proteins play important roles in response to biotic and abiotic stresses in poplar [36]. However, few reports of PPRs involved in these stresses in rice have been documented. In this study, to determine their potential roles to respond to various environmental stresses, we investigated the expression profiles of rice *PPR* genes under different treatments using high-throughput sequencing data. These stresses included rice stripe virus (RSV), bacterial blight disease, rice blast and phosphorus, cadmium, and cold stresses as well as drought and salinity stresses (Fig. 6). Our expression profiling results showed that many *PPR* genes were induced under the stresses, especially in the bacterial blight disease, cold and cadmium stresses (Fig. 6b, f, and g). Interestingly, some PPRs, including *LOC_Os02g46980* and *LOC_Os07g09370* were differentially expressed in both cold, drought, salt stresses and RSV, rice blast infection, respectively (Fig. 6 and Additional file 1: Table S5), indicating that these *PPR* genes might participate in many stress response processes in rice.

### Expression regulation of *PPR* genes under salt and drought stresses

**Fig. 7:**
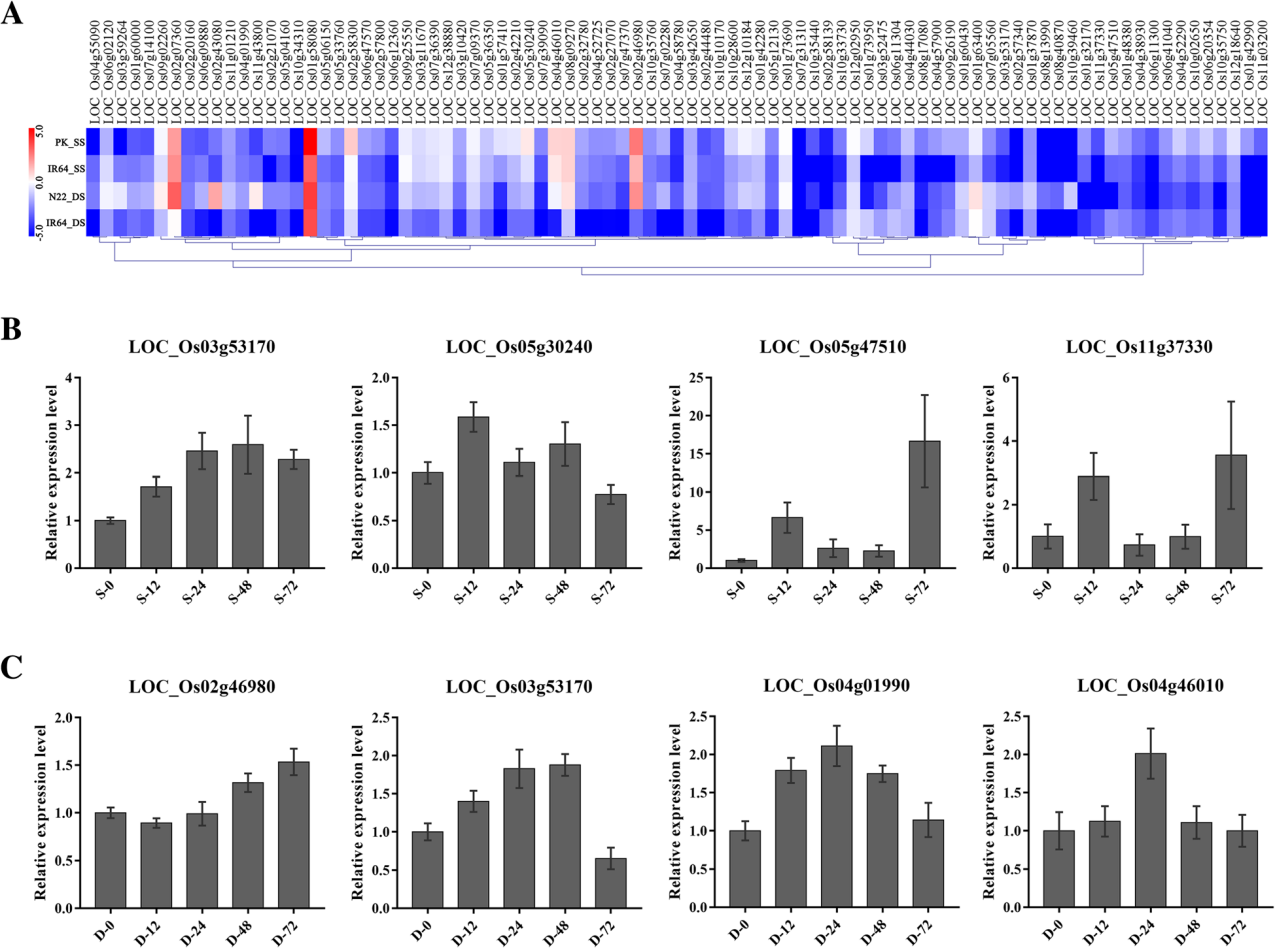
Expression patterns of *PPR* genes under the salt and drought salt stresses. **(a)** Heatmap showing different expression profiles of *PPR* genes in drought-tolerant (Nagina 22, N22) and salinity-tolerant (Pokkali, PK) rice cultivars with IR64 (susceptible cultivar) under the salt stress (SS) and drought stress (DS). **(b)** Expression levels of 4 genes under salt condition (S) after 12 h, 24 h, 48 h and 72 h. **(c)** The expression level of 4 genes under drought condition (**d**)

To determine the response of *PPR* genes to abiotic stress, the RNA-seq data of rice subjected to the treatment of salt and drought stresses [42] were analyzed in the study. The results revealed that a total of 75 *PPR* genes were up-regulated (> 2) under salt stress conditions and 73 *PPR* genes were up-regulated under the drought stress conditions compared with the control (Fig. 7a). We randomly chose seven *PPR* genes and verified their expression patterns using qRT-PCR under salt and drought stresses (Fig. 7). Under salt stress condition, gene *LOC_Os05g30240* showed a slight up-regulated expression. Both gene *LOC_Os05g47510* and gene *LOC_Os11g37330* were more highly expressed at 24 h and 72 h after 200 mM NaCl treatment (Fig. 7b). Under drought stress [10% (*w*/*v*), PEG6000], the genes *LOC_Os02g46980* and *LOC_Os04g01990* showed significant up-regulation compared with the control, while *LOC_Os04g46010* exhibited increase expression only at 24 h (Fig. 7c). Those *PPR* genes might be involved in salt stress or drought stress responses. *LOC_Os03g53170* also showed obvious up-regulation expression under the two stresses condition, indicating that it might participate both in salt and drought stress tolerance.

**Fig. 8:**
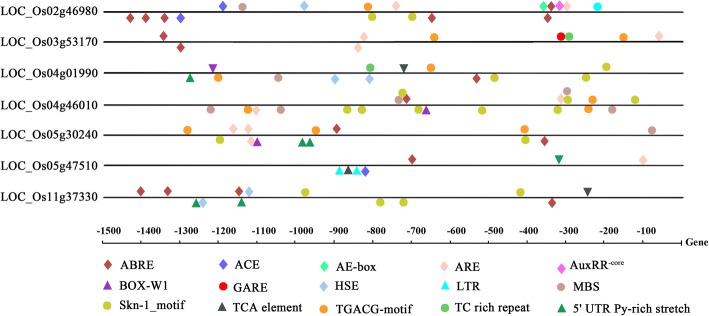
The promoter analysis in the seven *PPR* genes**.** Different *cis*-elements were indicated by different color symbols and placed in their relative position on the promoter. Symbols presented above the line indicate the elements at the forward strand, while those below indicate the reverse strand. The ABA-responsive element (ABRE), light response cis-acting element (ACE), light response module (AE-box), anaerobic induction element (ARE), auxin responsive element (AuxRR-core), fungal elicitor responsive element (BOX-W1), gibberellin-responsive element (GARE), heat shock element (HSE), low temperature responsive element (LTR), MYB-binding site (MBS), endosperm expression required element (Skn-1_motif), salicylic acid responsive element (TCA), Methyl jasmonate-responsive element (TGACG motif), defense and stress responsive element (TC-rich repeat), and element conferring high transcription level (5’ UTR Py-rich stretch) were analyzed

The *cis*-elements in promoter regions are closely associated with gene transcription and their response to stress. Therefore, 1.5 kb upstream sequences from the ATG initiation code were downloaded and analyzed using PlantCARE databases [43]. Some stress-responsive *cis*-acting elements were showed in promoter regions of the seven *PPR* genes, including ABRE, ARE, HSE, Skn-1_motif, TGACG motif and 5’ UTR Py-rich stretch (Fig. 8). All these elements had an important role in the regulation of gene transcription induced by biotic and abiotic stress. Among the seven PPR genes, the *LOC_Os05g47510* promoter only had seven elements, while *LOC_Os04g46010* had maximum20 elements. Almost every promoter region in the *PPR* genes contained ABRE, Skn-1_motif and TGACG motif. Although the correlation between *cis*-elements and response of genes in stresses conditions require more experimental investigation, these results revealed the stress-responsive nature of *PPR* genes.

## Discussion

The PPR protein family is a large family that has been identified in many land plants. Previous research has shown that there were 477 *PPR* genes in rice [33]. In the present study, we identified 491 *PPR* genes in the rice genome. Furthermore, to understand the function, structure, and evolution of these genes, we analyzed the gene distribution in the genome, the gene structure and the subcellular location, performed functional analysis as miRNA targets, constructed a phylogenetic tree, and performed the gene expression analysis under different stresses. Our results showed that the function of *PPR* genes is varied during the development and stress response in rice.

In our evolutionary analysis, PPR proteins family in rice can be divided into P and PLS subfamilies based on the arrangement of ten PPR motifs and one DYW motif. Phylogenetic analysis also showed distinct differences between the two groups of proteins, except that some PLS proteins mixed with the P subfamily (Fig. 2a). These findings were similar to the phylogenetic relationship of poplar, which also had two distinct subfamilies, except that 7 PLS proteins clustered into the P subfamily [36]. We analyzed the consensus sequences of ten PPR motifs, including 1 P motif and 9 P-like motifs (Fig. 2). There were extremely similar to the sequence of other species that were previously reported [15], which indicates the conservation of PPR proteins during the evolution among plants.

In the present study, gene structure analysis revealed that most *PPR* genes were intron-less; 65% of *PPR* genes contained no intron and 16% possessed one intron, while only 7% of *PPR* genes had 5 or more introns. In *Arabidopsis*, more than 80% of *PPR* genes contained a single exon, and only 12% contained one intron [14, 33]. The intron-less nature of the majority of *PPR* genes and the wide distribution of *PPR* genes on chromosomes in land plants may reveal duplication event of *PPR* genes. It had been presumed that *PPR* genes family was derived from amplification by retro-transposition [4, 14, 33]. Synteny relationship of the PPR proteins in rice revealed that 276 *PPR* genes had synteny with other *PPR* genes in rice. *LOC_Os11g01210* and *LOC_Os12g01210* shared more than 1000 bp of the same sequence in their coding sequences. These results could prove that “ancient” *PPR* genes with some introns were amplified by reverse transcription and integrated into the genome, which resulted in a large number of intron-less *PPR* genes in the genome with a wide distribution in all chromosomes.

Previous studies have shown that PPR proteins are RNA-binding proteins that play a major role in post-transcription via RNA editing, splicing, cleavage, stability or translation in mitochondria or plastids [1]. In maize, PPR10 proteins could stabilize RNAs by binding specific regions and blocking 5′ or 3′ degradation, which can promote translation efficiency. It could also activate translation of *atpH* RNA by preventing the formation of secondary structure and exposing ribosome-binding sites for translation [44]. It was hypothesized that maize PPR5 proteins prevent the formation of an RNA hairpin, which could mask a key cis-element for splicing of group II introns [45]. Several PPR-encoding Rf proteins induced the cleavage of sterility-associated mitochondrial RNAs. Those proteins induced the formation of adjacent 5′ and 3′ termini, which stimulate site-specific endonucleolytic cleavage [46]. PLS PPR proteins are almost exclusively associated with C-to-U RNA editing, which always alters the coding potential of the transcript. Although the precise mechanism of PRR editing activity is not well documented, it had been proved that multiple-organellar RNA-editing factor (MORF) proteins were required for editing with PPR proteins and other factors [47].

PPR proteins could be the target genes of some miRNAs that regulate in abiotic stress responses and fertility restore [40]. A total of 49 miRNAs were predicted to target 64 *PPR* genes in this study (Additional file 1: Table S3). One target of osa-miR1425 is the *Rf-1* gene, which not only leads to an increase in the number of potentially fertile pollen grains but also enhances cold tolerance in hybrid rice [48, 49]. In our study, we validated three *PPR* genes targeted by corresponding miRNAs (osa-miR1862d, osa-miR396a and oss-miR444b.2) (Fig. 5). Compared to the WXS (F) cultivars, osa-miR1862d and oss-miR444b.2 were both up-regulated in P2 (mother cell formation stage) and P3(meiosis stage) of the WXS (S). osa-miR396a appeared to be up-regulated in both P2 and P3 stage of WXS (S) [39]. The expression changes of the miRNAs in the same stage of fertile and sterile rice were consistent with potential functions of target *PPR* genes acting as fertility restorers. The results suggested that miRNAs and their target *PPR* genes might be related to male sterility and anther development during fertility transition.

In rice, dual-localized PPR protein *OsPGL1* was preferentially expressed in leaves particularly in the four and five leaf and was essential for the chloroplast development in rice leaves [47]. A fertility restorer gene, *Rf4*, was expressed at the highest level in the anthers at the tri-cellular pollen stage, where the fertility restoration occurred (Table 2). Therefore, gene expression patterns would be able to reveal important clues for studying genes function. In this study, analysis of public data showed that *PPR* genes in rice have temporal and spatial expression patterns. The results of real-time PCR showed that some *PPR* genes, including *LOC_Os03g19650*,*LOC_Os04g49350*, *LOC_Os05g28500* and *LOC_Os12g01210* were predominantly or specifically expressed in young leaves (Fig. 6). In addition, the four genes were all predicted to locate in chloroplasts, indicating that they may be involved in leaf development. The genes *LOC_Os01g12810*, *LOC_Os04g14130* and *LOC_Os12g44170* were also highly expressed at the stage of panicles and seed development. We inferred that those genes might participate in embryonic and seed development.

**Table 2:** Some *PPR* genes reported in rice

PPR Genes	MSU_Locus	localization	Function of gene	Phenomenon of Mutation	References
*OsPPR1*	LOC_Os09g24680	chloroplast	play a role in the chloroplast biogenesis	albinism and lethality	Gothandam et al. Plant Mol Biol (2005)
*WSL*	LOC_Os01g37870	chloroplast	affects chloroplast development and abiotic stress response	displays chlorotic striations early in development and enhanced sensitivity to ABA, salinity, and sugar	Tan et al. Mol Plant (2004)
*OsPPR6*	LOC_Os05g49920	chloroplast	regulates early chloroplast biogenesis and participates in editing of *ndhB* and splicing of *ycf3* transcripts	albino leaves and seedling death	Tang et al. Plant Mol Biol (2017)
*OspTAC2*	LOC_Os03g60910	chloroplast	plays a critical role in chloroplast development	albino and seedling-lethal	Wang et al. J Genet Genomics (2016)
*OsV4*	LOC_Os04g39970	chloroplast	plays an important role during early chloroplast development under cold stress	albino phenotype and abnormal chloroplasts at 20 °C green leaves and normal chloroplasts at 32 °C	Gong et al. J Integr Plant Biol (2013)
*OGR1*	LOC_Os12g17080	mitochondria	essential for RNA editing required for normal growth	opaque seeds, delayed seed germination, retarded growth, dwarfism and sterility	Kim et al. Plant J (2009)
*Rf1a/ Rf5*	LOC_Os10g35436	mitochondria	restore the fertility via cleavage of *atp6*/*orf79* mRNA.	cannot restore male fertility in BT-CMS	Wang et al. Plant Cell (2002)
*Rf4*	LOC_Os10g35240	mitochondria	reduce the expression of *orf352* and restore the fertility of WA-CMS	cannot restore male fertility in WA-CMS	Tomohiko et al. Rice (2014)
*OsPGL1*	LOC_Os12g06650	dual-located in mitochondria and chloroplast	involved in both chloroplast RNA editing of *ndhD-878* and mitochondrial RNA editing of *ccmFc-543*	exhibited pale green leaves at all vegetative stages	Xiao et al. J Exp Bot (2018)
*PPS1*	LOC_Os12g36620	mitochondria	required for specific editing sites in *nad3* in mitochondria	delayed development and partial pollen sterility	Xiao et al. New Phytol (2018)

In previous studies, *PPR* genes changed their expression under biotic and abiotic stress and regulated growth in many plants. In rice, *WSL* is localized to chloroplast and involved in abiotic stress response. *Wsl* mutant showed enhanced sensitivity to ABA, salinity, and sugar [50]. Another PPR protein OsV4 plays an important role during early chloroplast development under cold stress [31]. In this study, we analyzed the expression patterns of *PPR* genes under different biotic and abiotic stresses (Fig. 7). The results showed that many *PPR* genes were induced by stresses. Some *PPR* genes had clearly up-regulation expression under phosphorus and cold stresses. The genes *LOC_Os02g46980* and *LOC_Os07g09370* were found differentially expressed in cold, drought, salt stresses and RSV, rice blast infection, respectively (Additional file 1: Table S5), indicating that these *PPR* genes might participate in many processes and respond to different stresses in rice. Further qRT-PCR analysis validated that some genes were up-regulated under salt or drought stress (Fig. 7). The genes *LOC_Os05g30240*, *LOC_Os05g47510* and *LOC_Os11g37330* were induced by salt stress, while *LOC_Os02g46980*, *LOC_Os04g01990* and *LOC_Os04g46010* were highly expressed under drought than in normal conditions. *LOC_Os03g53170* had a significantly increased expression under both stresses.

Many studies showed that cis-elements were involved in the expression of genes under different stresses. In *Arabidopsis*, three NAC transcription factors bound to drought-responsive cis-element (MYC-like sequence) in *ERD1* promoter and improved its drought stress tolerance [51]. A *cis*-acting element in the 5′-regions of the *cor15a* (cold-regulated) gene could be activated in response to low temperature, which was important for plants in cold resistance [52]. In our study, *cis*-element analysis results revealed that ABRE, Skn-1_motif and TGACG motif were found in the promoter regions of *PPR* genes that had a higher expression under salt and drought stresses. Although the mechanism should be further explored, the preliminary analyses of these elements would be helpful for understanding the gene responses to different stresses.

## Conclusions

In this study, we identified 491 rice PPR genes, which can be divided into four subgroups. Gene structure and distribution analysis showed that most *PPR* genes are intron-less and widely distributed across all rice chromosomes. PPR proteins were predicted to be located in chloroplasts or mitochondria, where they were involved in the post-transcriptional modification. We also analyzed their phylogenetic and syntenic relationships, their functions as miRNA targets, and their expression patterns in different issues in rice. Furthermore, the expression patterns during different stages and expression profiles under salt and drought stress treatment were also elucidated and validated. The result suggested that *PPR* genes might play roles in rice response to environmental stresses. Taken together, our data will provide insight into the further study of *PPR* genes function in rice.

## Methods

### Plant materials and stress treatments

Rice (*Oryza sativa L. japonica* cv. Nipponbare) seeds were grown in containers with sponges as supporting materials in sterile water at 28 °C with 24 h light. After 7 days, the seedlings were transferred into a paddy at Wuhan University (30°34’ N; 114°17′ E) under natural conditions. Plant materials for expression analysis were: (i) 7-day-old leaves (shoot), 15-day-old leaves (seedling) and 20-day-old leaves (20-L); (ii) panicle before emergence inflorescence and after emergence inflorescence, 5–10 cm panicles (P3) and 10–15 cm panicle (P4); (iii) 5 DAP and 10 DAP seeds.

The 14-day seedlings cultivated in containers were separately treated with two stress conditions: salt stress (200 mM NaCl) and drought stress [20% (*w*/*v*)] (PEG 6000). Leaves of the treated samples were collected at 0, 12, 24, 48, and 72 h. All materials were taken, quickly frozen in liquid nitrogen, and stored at − 80 °C until RNA extraction.

### Database screening and identification of *PPR* genes

Some databases were used to search *PPR* genes in rice. First, BLASTP searches of the PPR domain, “PF01535” [4], was performed on the website of the Rice Genome Annotation Project (RGAP, http://rice.plantbiology.msu.edu/) to find *PPR* genes. Second, ‘Pentatricopeptide repeat’ was used as a keyword in a functional annotation search at the Rice Annotation Project Database (RAP-DB, http:// rapdb.dna.affrc.go.jp/). The results of the two searches were integrated and then redundant genes were discarded. Finally, the protein sequences were submitted to the PPR Database (http://www.plantppr.com/) [15] and protein structures were automatically analyzed. According to the results, proteins that had no PPR motif or only one PPR motif were discarded.

### Gene distribution and structure analysis

All PPR gene loci were searched in the RDAP and DAP-DB database and their information including chromosome location and position. The map of genes distributed across chromosomes was created with Mapchart software in the study.

For gene structure analysis, the exon and intron structures of individual *PPR* genes were illustrated using the Gene Structure Display Server (GSDS; http://gsds.cbi.pku.edu.cn/ ) by aligning the genomic DNA sequences with the corresponding cDNA sequences from the RADP and RAP-DB database.

### Consensus sequence of PPR motif analysis

There were 10 PPR motifs in PPR proteins including one P motif and nine PPR-like motifs (P1, P2, L1, L2, S1, S2, SS, E1 and E2). All sequences of each motif were analyzed based on PPR proteins sequence and structure. The consensus sequence and distribution of amino acid residues at the corresponding positions in the PPR motif were generated using the WebLogo program with default parameters (http://weblogo.berkeley.edu/logo.cgi).

### Phylogenetic and synteny analysis

Multiple sequence alignment of 491 PPR proteins from rice and 48 PPR proteins from other species, including *Arabidopsis*, moss, tomato and foxtail millet, was conducted using the MUSCLE method. A phylogenetic tree was conducted by the neighbor-joining (NJ) method with MEGA 7.0 [53] and bootstrap analysis of 1000 replicates.

All rice PPR protein sequences were searched against themselves using the BLASTp program with the E-value setting to 1e-10. Then, the result file and the GFF files of the rice genome were inputted into software MCScanX to analyze the syntenic relationship [54] and visualized using CIRCOS (http://circos.ca/).

### Expression patterns of *PPR* genes in various tissues and different stresses

To study expression patterns of rice *PPR* genes, the RNA-seq data were downloaded from the NCBI and RGAP. These data contained a wide range of developmental stages of rice, including shoots, leaves-20 days, pre and post-emergence inflorescence, anther, pistil, seed-5 DAP (Day After Pollination), seed-10 DAP, embryo-25 DAP, and endosperm-25 DAP. The gene expression data under different stresses(phosphate stress, cold stress, drought/salinity stress and rice stripe virus stress) were downloaded from NCBI (SRA097415, SRP026336, GSE60287, GSM1921841- GSM1921846) [42, 55–57] and DDBJ (DNA Data Bank of Japan) Sequence Read Archive (DRA001092) [58]. In addition, the RNA-seq data of rice under blast infection and bacterial blight disease were downloaded from public data [59, 60]. The RNA-seq data were reanalyzed and were log2 transformed. Heat map representing hierarchical clustering was created by MeV4.9 (MultiExperiment Viewer) software with the log-transformed values.

### Stem-loop RT-PCR and real-time PCR analysis

Total RNA was isolated using TRIzol (Takara, Dalian, China) reagent from collected samples. RNase-free DNase was used to degrade DNA from total RNA at 37 °C for 30 min. For mRNA reverse transcriptions, the first strand cDNA derived from mRNA was synthesized from 1 μg total RNA using RevertAid First Strand cDNA Synthesis Kit (Fermentas, USA) according to the manufacturer’s instructions. For miRNA validation, 1 μg of total RNA was reverse-transcribed using miRNA-specific stem-loop primers for reverse transcription of miRNA. The reactions were incubated for 30 min at 16 °C, followed by 60 cycles of 30 °C for 30 s, 42 °C for 30 s and 50 °C for 1 s. The reactions were terminated by heating at 70 °C for 5 min. All stem-loop primers were designed according to Varkonyi-Gasic et al. [61].

Quantitative real-time PCR (qRT-PCR) was carried out by SYBR-green fluorescence with an ABI StepOnePlus Real-Time PCR System. U6 snRNA and *β-actin* were used as internal control for miRNA and mRNA qRT-PCR analysis, respectively. All cDNAs were diluted 10 times and 1 μl diluted product was mixed with 5 μl of 2 × SYBR reaction mix and 0.2 μM primers in a 10 μl volume reaction system. The PCR conditions were 30 s at 95 °C, followed by 40 cycles of 10 s at 95 °C, 30 s at 56 °C and 15 s at 72 °C. Four replicates were performed for each sample. After the amplification, the melting curve was determined for specific product. Three biological replicates for each sample were performed for each sample. The relative expression levels were calculated using a ^△△^CT method and the melting curve was carried out for each PCR product to avoid nonspecific amplification. All primers used in this study are listed in Additional file 1: Table S6.

### Promoter sequence analysis for potential *cis*-regulatory elements

To analyze *cis*-elements in the PPR gene promoters, 1.5 kb 5′ upstream region sequences were downloaded from the RAP-DB database. The sequences were analyzed using PlantCARE databases (http://bioinformatics.psb.ugent.be/webtools/plantcare/html/) to find the potential *cis*-acting regulatory elements.

### Subcellular localization of one PPR protein

All protein sequences were analyzed by TargetP 1.1 [37] and Predotarv.1.04 [38] to predict their subcellular localization in the study. To verify the subcellular localization of PPR proteins, the complete CDS without stop codons were amplified and inserted in front of the green fluorescence protein (GFP) coding sequence in the HPT-GFP vector. The PPR-GFP fusion proteins were expressed under the control of the CaMV 35S promoter in rice protoplasts. GFP fluorescence was visualized with a confocal laser scanning microscope with excitation wave lengths at 488 nm and emission wave lengths at 509 nm. The mitochondria were stained with MitoTracker Red CMXRos (Invitrogen, Carlsbad, CA, USA), whose excitation and emission wave lengths are 579 and 599 nm, respectively.

## Additional files

Additional file 1: **Table S1.** The general information of *PPR* genes in rice genome. **Table S2.** *PPR* genes subcellular location results in rice by TargetP and Predotar. **Table S3.** *PPR* genes as miRNA targets in rice. **Table S4.** FPKM values of *PPR* genes in rice various organs and tissues. **Table S5.** RNA-seq data of *PPR* genes under different biotic and abiotic stresses. **Table S6.** Primers used in the study. (ZIP 805 kb)

Additional file 2: **Figure S1.** Structures of PPR proteins and consensus sequence of PPR motif in rice. **(A)** Typical structures of PPR proteins in rice. The number of motifs in each protein can vary from 2 to 28 in rice. **(B)** The consensus sequence of 10 PPR motif in rice. The overall height of each stack indicates the conservation of the sequence at that position and the bit score indicates the relative frequency of the corresponding acid. The lengths of the motifs can be estimated using the scale at the bottom. **Figure S2.** Distribution of PPR genes in rice chromosomes. The 491 *PPR* genes are widely and unevenly distributed on all 12 chromosomes of rice. **Figure S3.** Exon/intron structures of the PPR genes in rice. Yellow boxes represent exons, and black lines represent introns. Upstream or downstream regions are indicated by blue boxes. The sizes of exons and introns can be estimated using the scale at the bottom. **Figure S4.** Phylogenetic relationship of the PLS subgroup genes in rice and other species. Evolutionary relationships of PLS subgroup genes from rice, Arabidopsis, moss, tomato, and foxtail millet. The genes whose IDs start with LOC represent rice gene, AT represents Arabidopsis, Pp represents moss, Solyc represents tomato, and Seita represents foxtail millet. **Figure S5.** Expression patterns of PPR genes in different rice tissues. The FPKM expression values from RGAP of *PPR* genes at various developmental stages were log2 transformed, and a heat map was generated using the MeV4.9 software. Samples are indicated at the top of each lane: shoots, leaves-20 days, pre/post-emergence inflorescence (pre-EI, post-EI), anther, pistil, seed-5 DAP, seed-10 DAP, embryo- 25 DAP and endosperm- 25 DAP. The color scale (representing average log2 signal values) is shown at the top. (ZIP 6622 kb)

## Abbreviations

CDS: Coding sequence
CMS: Cytoplasmic male sterility
CRISPR: Clustered regularly interspaced short palindromic repeats
FPKM: Fragments per kilobase of transcript per million mapped reads
GFP: Green fusion proteins
MCScanX: Multiple collinearity scan toolkit
MeV: MultiExperiment viewer
miRNA: MicroRNA
NCBI: National Center for Biotechnology Information
PPR: Pentatricopeptide-repeat
qRT-PCR: Quantitative real-time PCR
RAP-DB: Rice annotation project database
RF: Restorer of fertility
RGAP: Rice genome annotation project
