# Supplementary figures and images for: Genome-wide analysis of the rice PPR gene family and their expression profiles under different stress treatments
Source: BMC Genomics. 2018 Oct 1;19:720. doi: 10.1186/s12864-018-5088-9 (PMC6167770; doi:10.1186/s12864-018-5088-9)

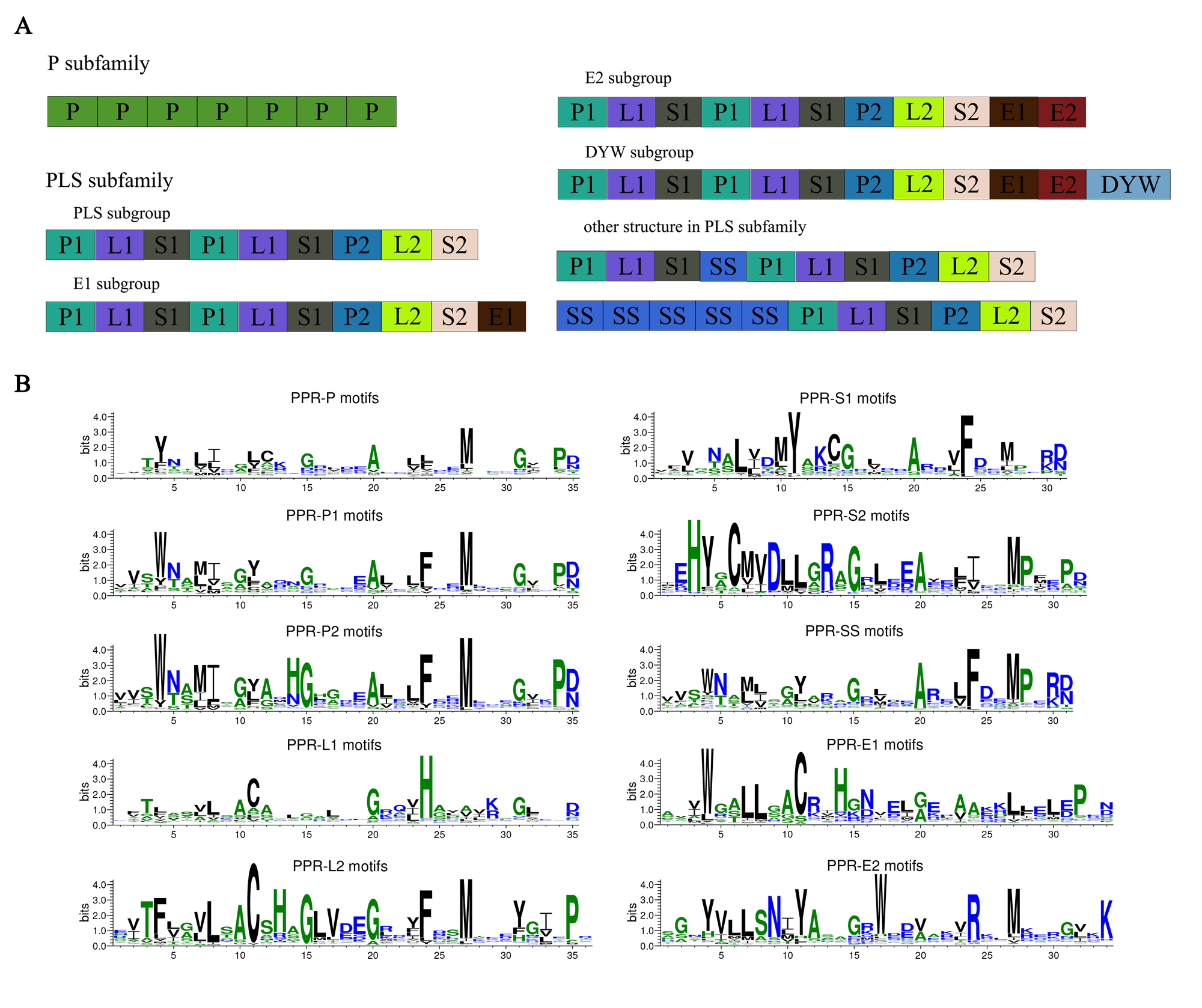

Supplement: Supplementary file 2 — Figure S1. Structures of PPR proteins and consensus sequence of PPR motif in rice. (A) Typical structures of PPR proteins in rice. The number of motifs in each protein can vary from 2 to 28 in rice. (B) The consensus sequence of 10 PPR motif in rice. The overall height of each stack indicates the conservation of the sequence at that position and the bit score indicates the relative frequency of the corresponding acid. The lengths of the motifs can be estimated using the scale at the bottom. Figure S2. Distribution of PPR genes in rice chromosomes. The 491 PPR genes are widely and unevenly distributed on all 12 chromosomes of rice. Figure S3. Exon/intron structures of the PPR genes in rice. Yellow boxes represent exons, and black lines represent introns. Upstream or downstream regions are indicated by blue boxes. The sizes of exons and introns can be estimated using the scale at the bottom. Figure S4. Phylogenetic relationship of the PLS subgroup genes in rice and other species. Evolutionary relationships of PLS subgroup genes from rice, Arabidopsis, moss, tomato, and foxtail millet. The genes whose IDs start with LOC represent rice gene, AT represents Arabidopsis, Pp represents moss, Solyc represents tomato, and Seita represents foxtail millet. Figure S5. Expression patterns of PPR genes in different rice tissues. The FPKM expression values from RGAP of PPR genes at various developmental stages were log2 transformed, and a heat map was generated using the MeV4.9 software. Samples are indicated at the top of each lane: shoots, leaves-20 days, pre/post-emergence inflorescence (pre-EI, post-EI), anther, pistil, seed-5 DAP, seed-10 DAP, embryo- 25 DAP and endosperm- 25 DAP. The color scale (representing average log2 signal values) is shown at the top. (ZIP 6622 kb) [file 12864_2018_5088_MOESM2_ESM.zip › Figure S1.tif]

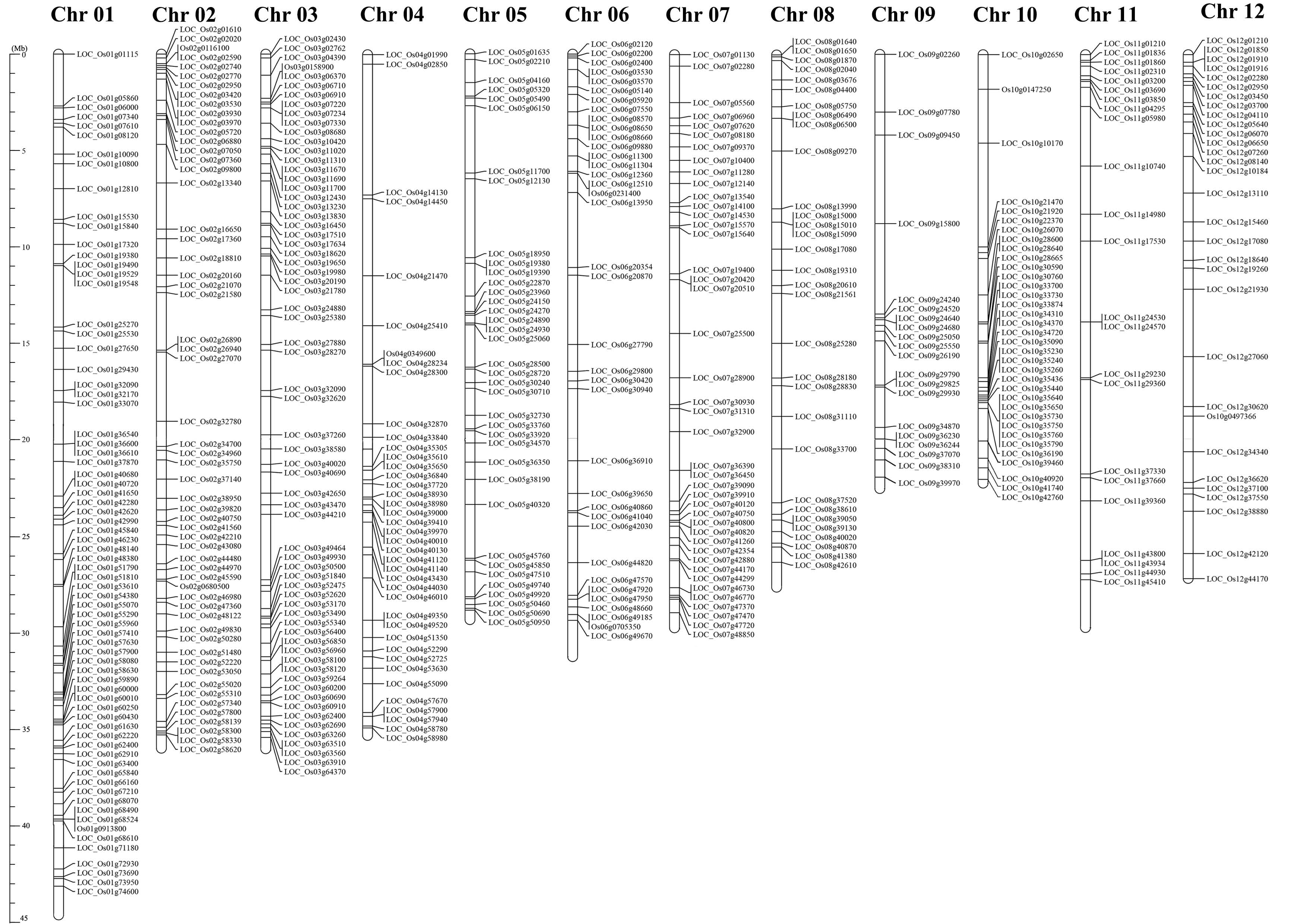

Supplement: Supplementary file 2 — Figure S1. Structures of PPR proteins and consensus sequence of PPR motif in rice. (A) Typical structures of PPR proteins in rice. The number of motifs in each protein can vary from 2 to 28 in rice. (B) The consensus sequence of 10 PPR motif in rice. The overall height of each stack indicates the conservation of the sequence at that position and the bit score indicates the relative frequency of the corresponding acid. The lengths of the motifs can be estimated using the scale at the bottom. Figure S2. Distribution of PPR genes in rice chromosomes. The 491 PPR genes are widely and unevenly distributed on all 12 chromosomes of rice. Figure S3. Exon/intron structures of the PPR genes in rice. Yellow boxes represent exons, and black lines represent introns. Upstream or downstream regions are indicated by blue boxes. The sizes of exons and introns can be estimated using the scale at the bottom. Figure S4. Phylogenetic relationship of the PLS subgroup genes in rice and other species. Evolutionary relationships of PLS subgroup genes from rice, Arabidopsis, moss, tomato, and foxtail millet. The genes whose IDs start with LOC represent rice gene, AT represents Arabidopsis, Pp represents moss, Solyc represents tomato, and Seita represents foxtail millet. Figure S5. Expression patterns of PPR genes in different rice tissues. The FPKM expression values from RGAP of PPR genes at various developmental stages were log2 transformed, and a heat map was generated using the MeV4.9 software. Samples are indicated at the top of each lane: shoots, leaves-20 days, pre/post-emergence inflorescence (pre-EI, post-EI), anther, pistil, seed-5 DAP, seed-10 DAP, embryo- 25 DAP and endosperm- 25 DAP. The color scale (representing average log2 signal values) is shown at the top. (ZIP 6622 kb) [file 12864_2018_5088_MOESM2_ESM.zip › Figure S2.tif]

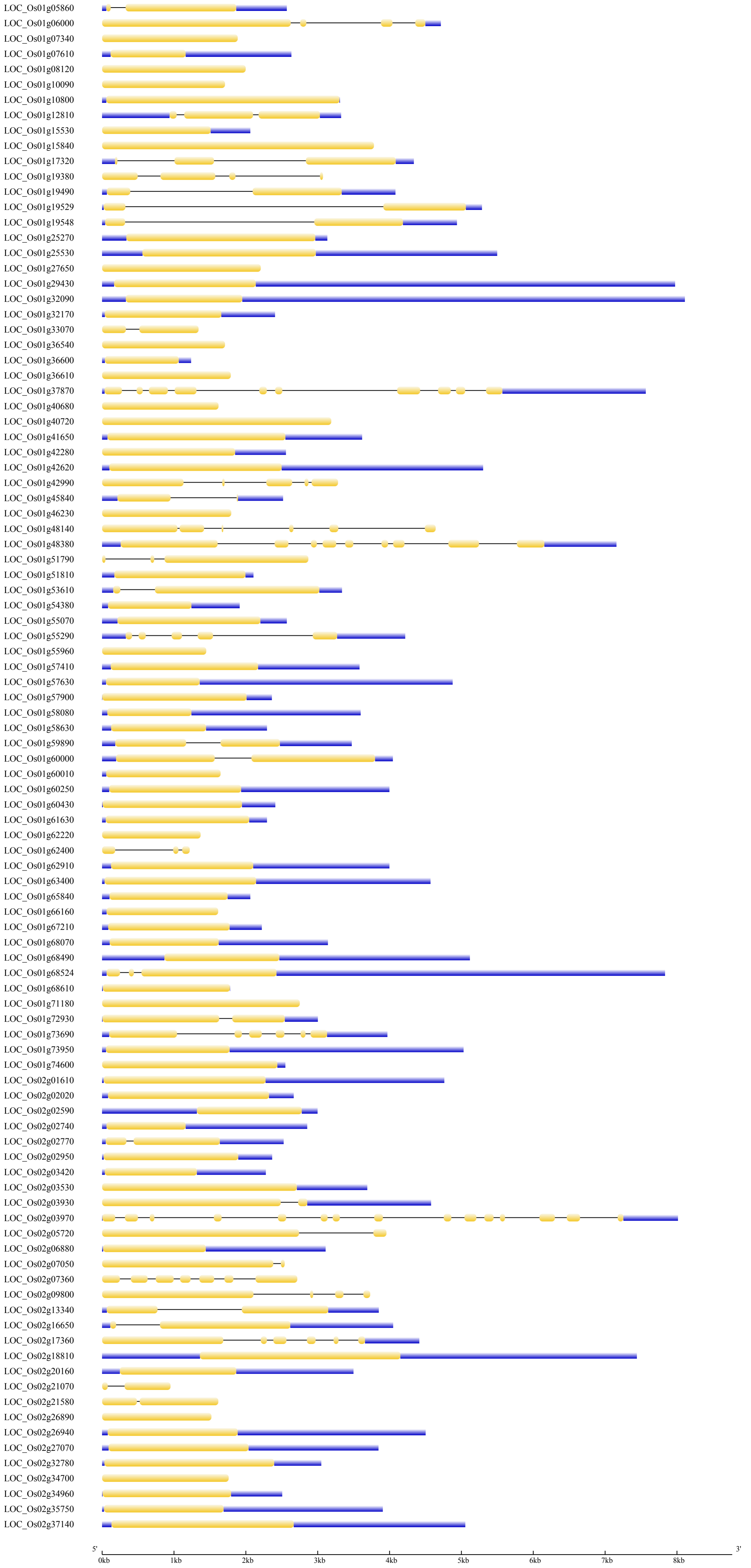

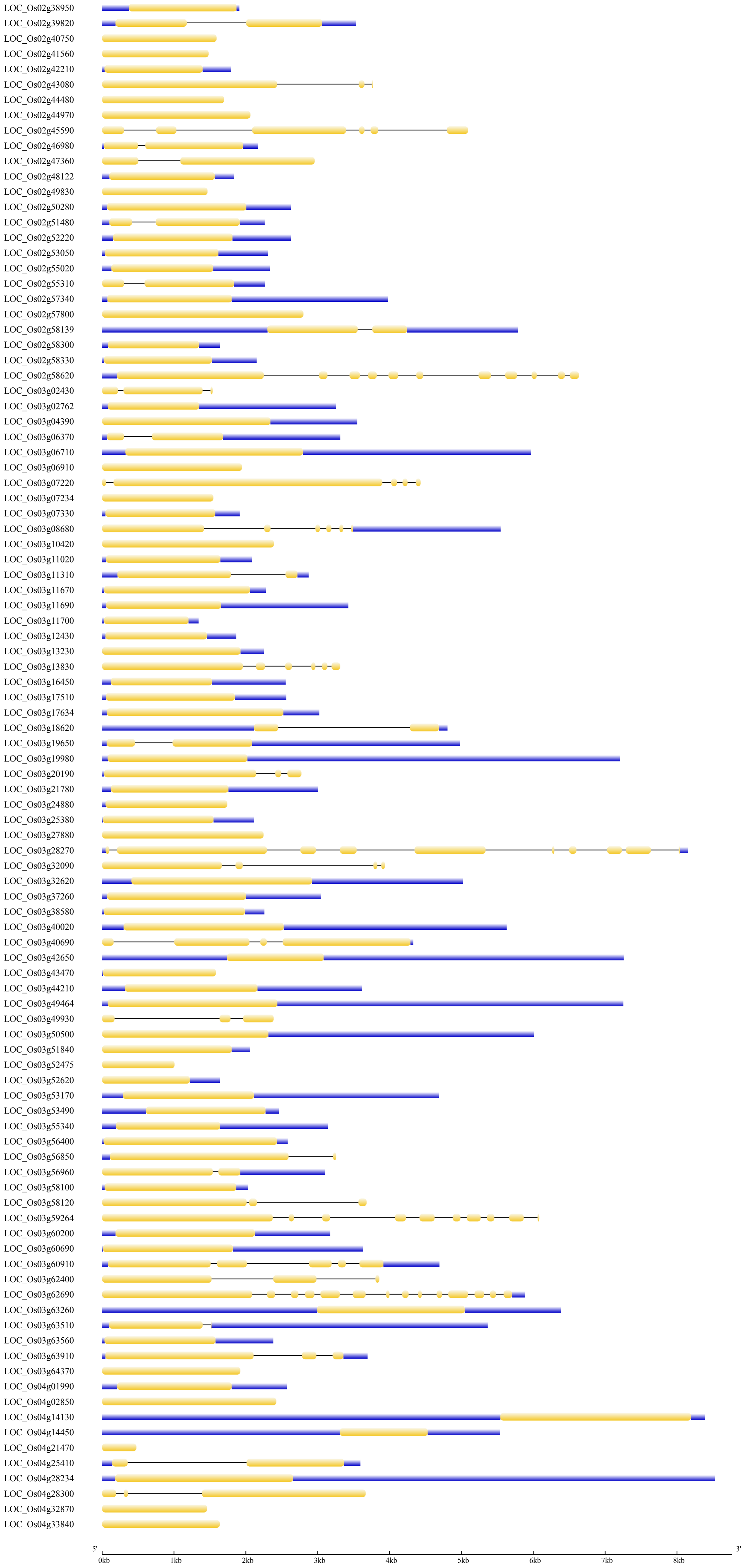

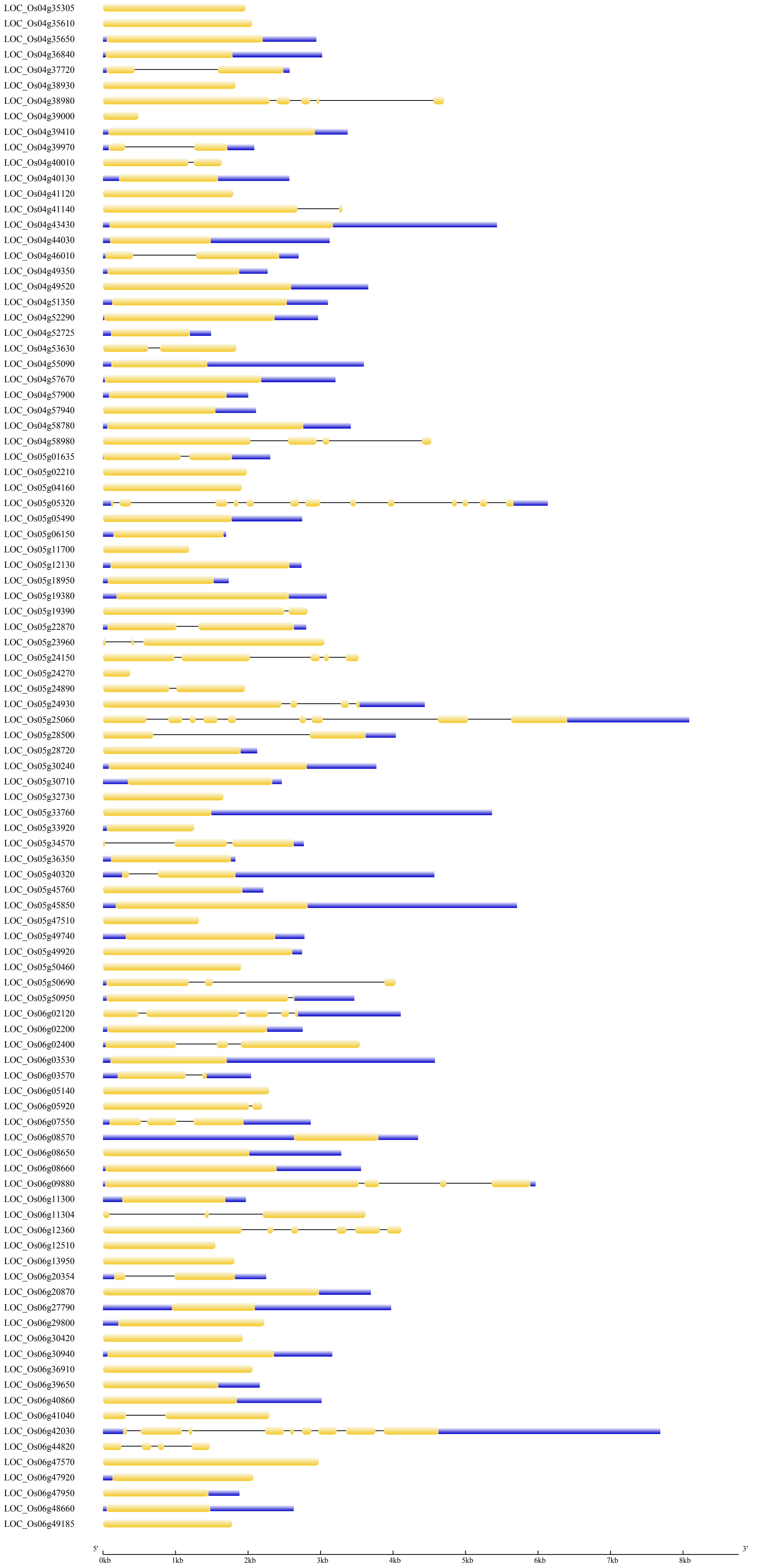

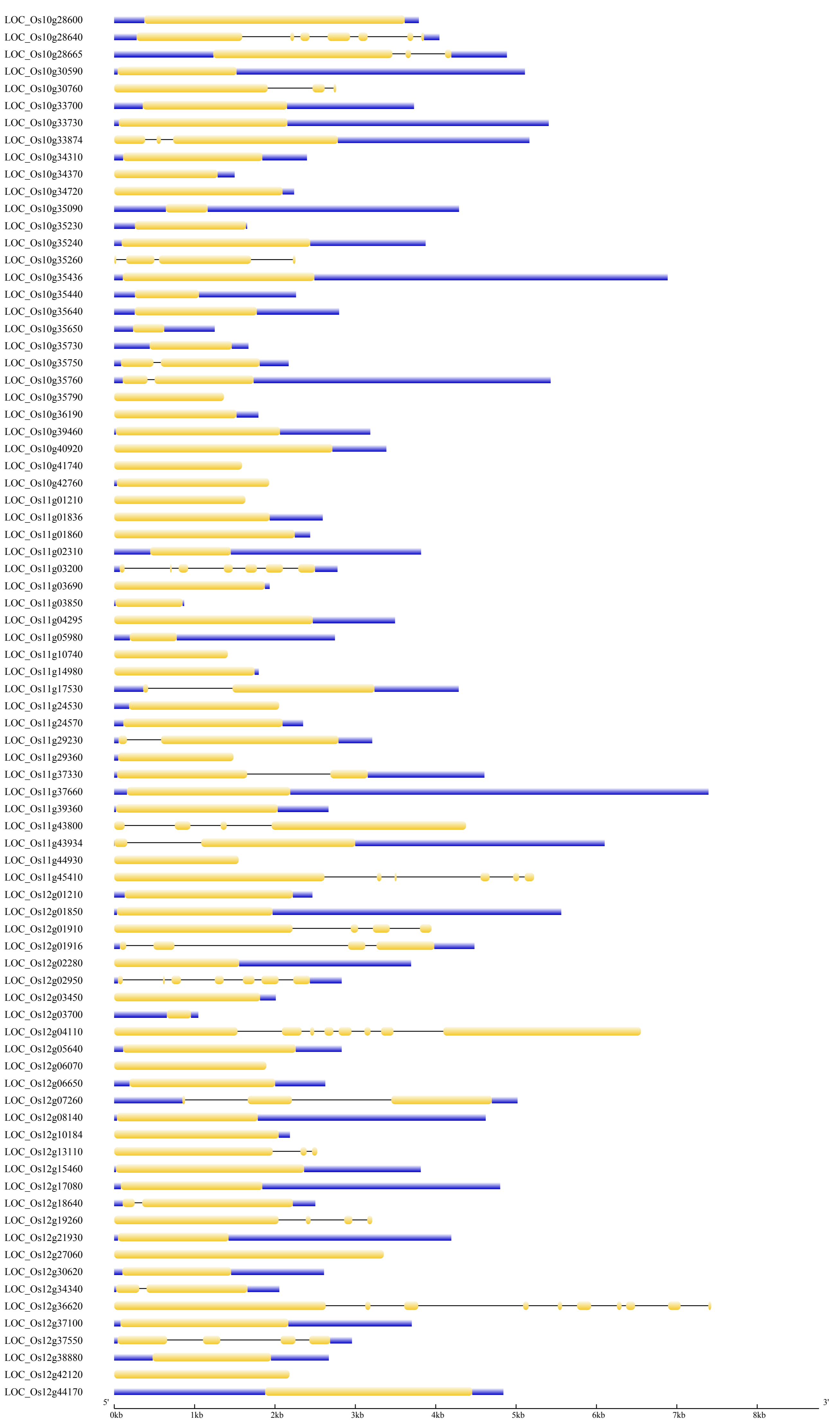

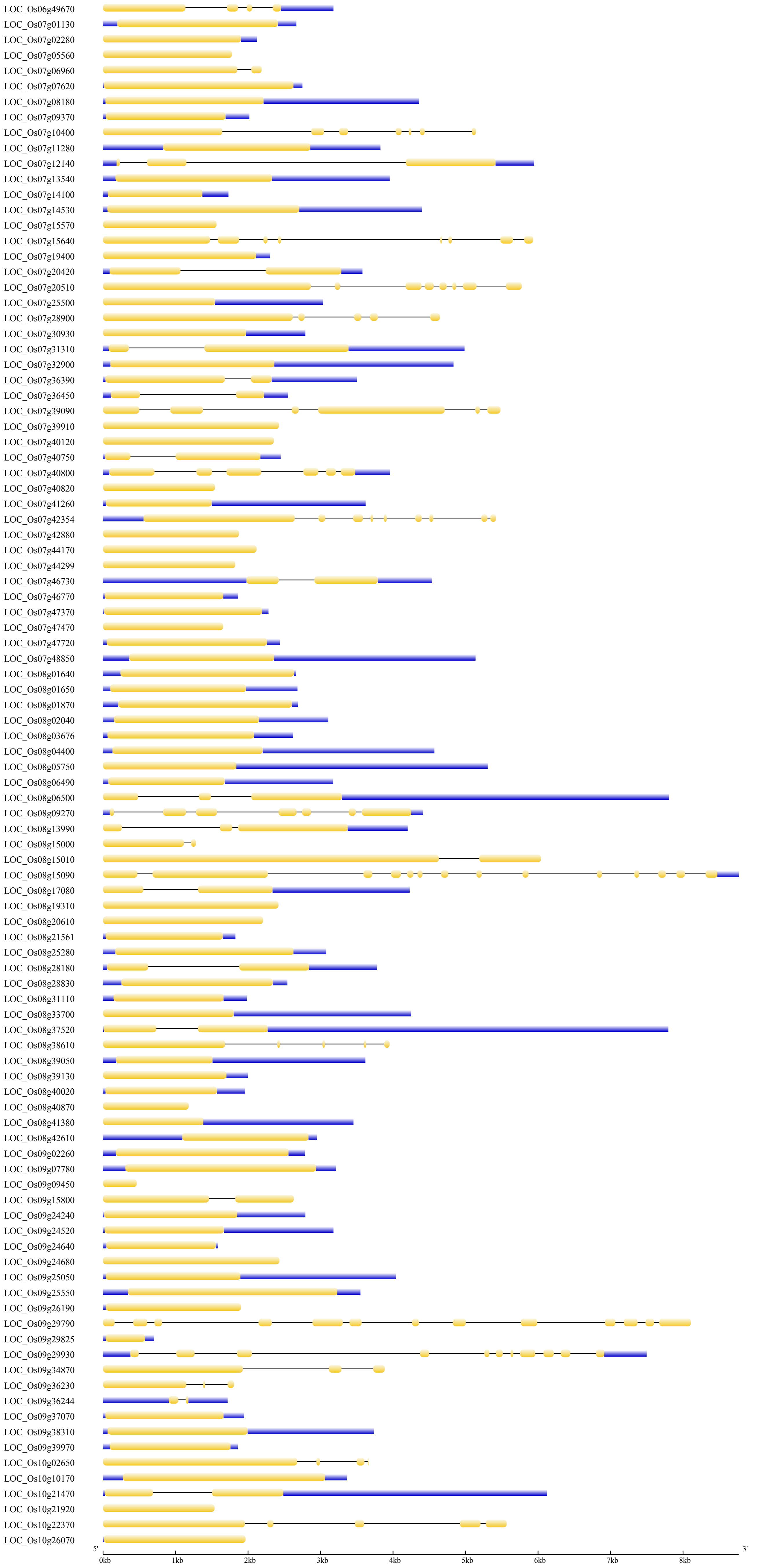

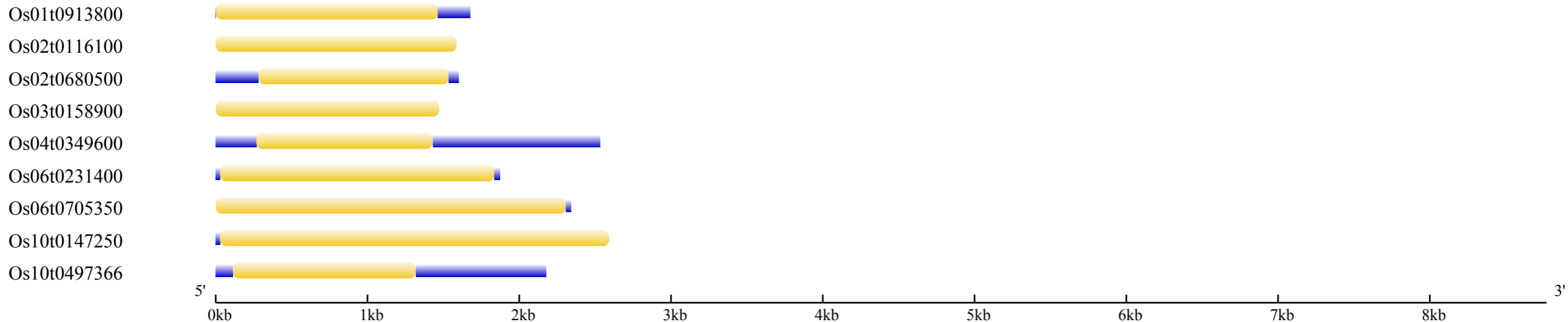

Legend: 491 *PPR* genes structure analysis

Yellow CDS    Blue upstream/ downstream    — Intron

Supplement: Supplementary file 2 — Figure S1. Structures of PPR proteins and consensus sequence of PPR motif in rice. (A) Typical structures of PPR proteins in rice. The number of motifs in each protein can vary from 2 to 28 in rice. (B) The consensus sequence of 10 PPR motif in rice. The overall height of each stack indicates the conservation of the sequence at that position and the bit score indicates the relative frequency of the corresponding acid. The lengths of the motifs can be estimated using the scale at the bottom. Figure S2. Distribution of PPR genes in rice chromosomes. The 491 PPR genes are widely and unevenly distributed on all 12 chromosomes of rice. Figure S3. Exon/intron structures of the PPR genes in rice. Yellow boxes represent exons, and black lines represent introns. Upstream or downstream regions are indicated by blue boxes. The sizes of exons and introns can be estimated using the scale at the bottom. Figure S4. Phylogenetic relationship of the PLS subgroup genes in rice and other species. Evolutionary relationships of PLS subgroup genes from rice, Arabidopsis, moss, tomato, and foxtail millet. The genes whose IDs start with LOC represent rice gene, AT represents Arabidopsis, Pp represents moss, Solyc represents tomato, and Seita represents foxtail millet. Figure S5. Expression patterns of PPR genes in different rice tissues. The FPKM expression values from RGAP of PPR genes at various developmental stages were log2 transformed, and a heat map was generated using the MeV4.9 software. Samples are indicated at the top of each lane: shoots, leaves-20 days, pre/post-emergence inflorescence (pre-EI, post-EI), anther, pistil, seed-5 DAP, seed-10 DAP, embryo- 25 DAP and endosperm- 25 DAP. The color scale (representing average log2 signal values) is shown at the top. (ZIP 6622 kb) [file 12864_2018_5088_MOESM2_ESM.zip › Figure S3.pdf]

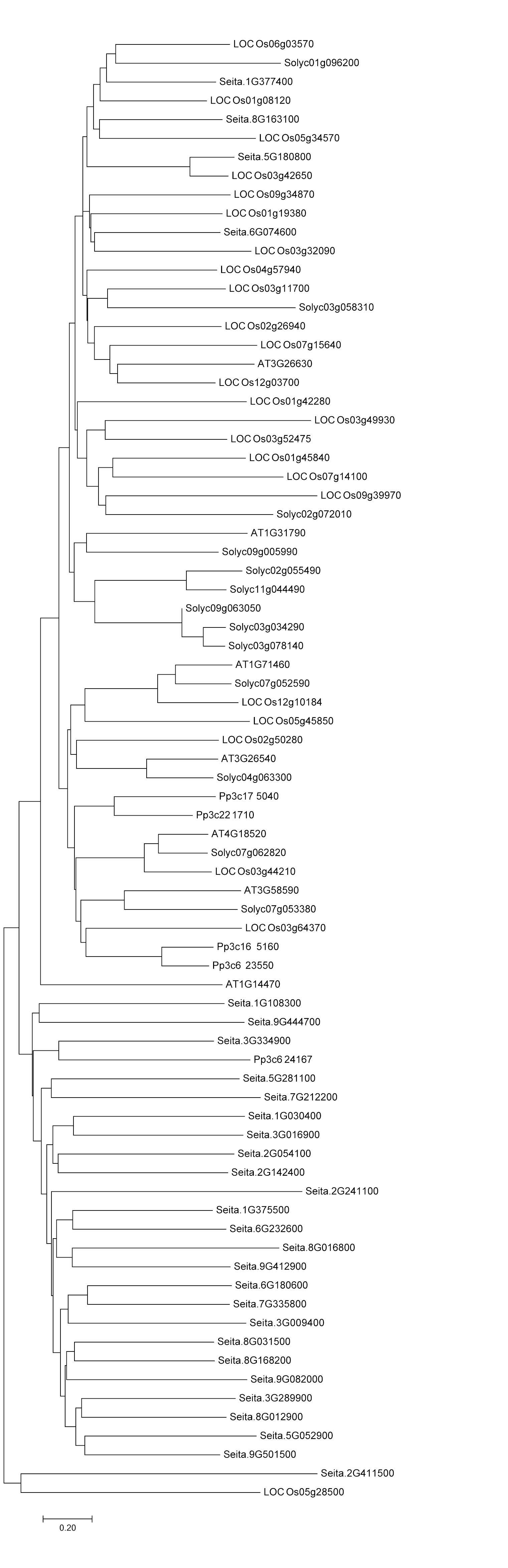

Supplement: Supplementary file 2 — Figure S1. Structures of PPR proteins and consensus sequence of PPR motif in rice. (A) Typical structures of PPR proteins in rice. The number of motifs in each protein can vary from 2 to 28 in rice. (B) The consensus sequence of 10 PPR motif in rice. The overall height of each stack indicates the conservation of the sequence at that position and the bit score indicates the relative frequency of the corresponding acid. The lengths of the motifs can be estimated using the scale at the bottom. Figure S2. Distribution of PPR genes in rice chromosomes. The 491 PPR genes are widely and unevenly distributed on all 12 chromosomes of rice. Figure S3. Exon/intron structures of the PPR genes in rice. Yellow boxes represent exons, and black lines represent introns. Upstream or downstream regions are indicated by blue boxes. The sizes of exons and introns can be estimated using the scale at the bottom. Figure S4. Phylogenetic relationship of the PLS subgroup genes in rice and other species. Evolutionary relationships of PLS subgroup genes from rice, Arabidopsis, moss, tomato, and foxtail millet. The genes whose IDs start with LOC represent rice gene, AT represents Arabidopsis, Pp represents moss, Solyc represents tomato, and Seita represents foxtail millet. Figure S5. Expression patterns of PPR genes in different rice tissues. The FPKM expression values from RGAP of PPR genes at various developmental stages were log2 transformed, and a heat map was generated using the MeV4.9 software. Samples are indicated at the top of each lane: shoots, leaves-20 days, pre/post-emergence inflorescence (pre-EI, post-EI), anther, pistil, seed-5 DAP, seed-10 DAP, embryo- 25 DAP and endosperm- 25 DAP. The color scale (representing average log2 signal values) is shown at the top. (ZIP 6622 kb) [file 12864_2018_5088_MOESM2_ESM.zip › Figure S4.tif]

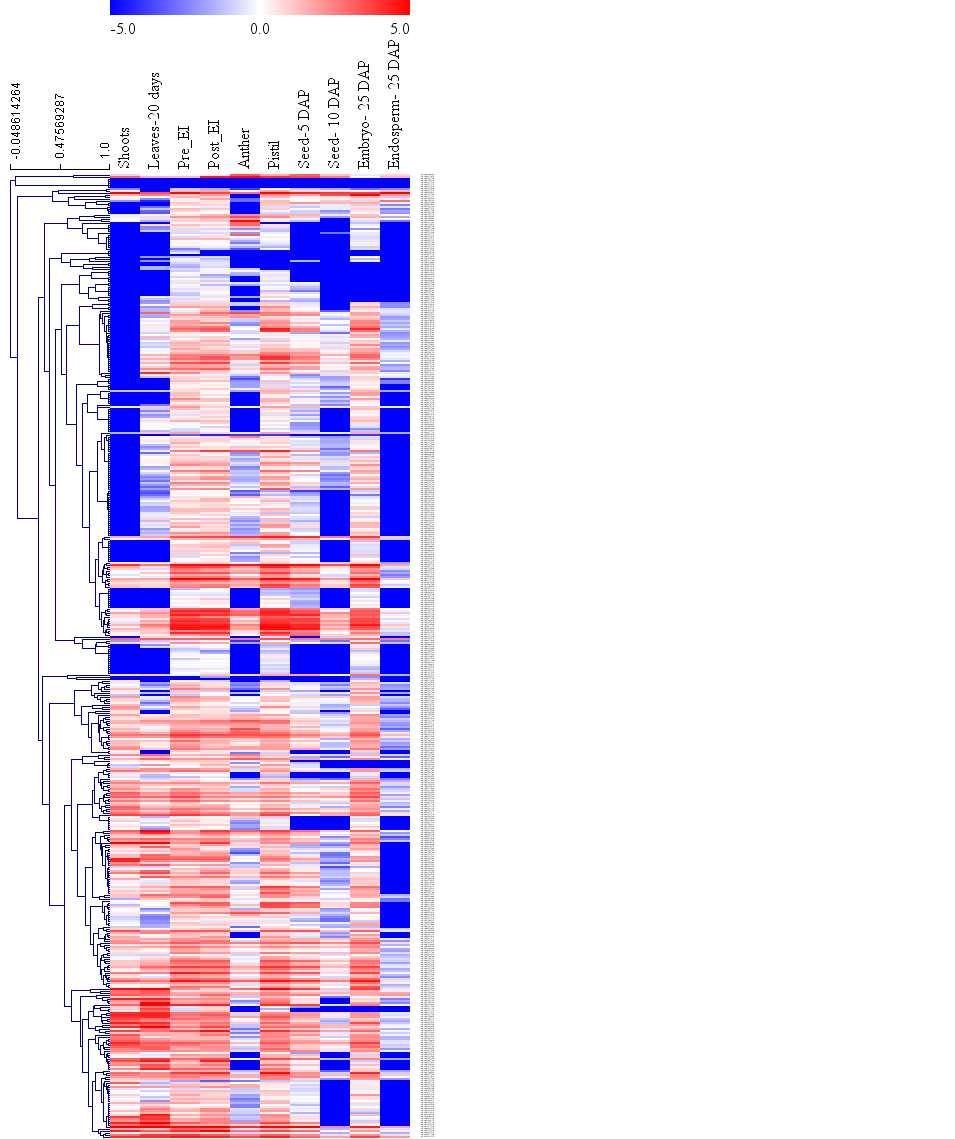

Supplement: Supplementary file 2 — Figure S1. Structures of PPR proteins and consensus sequence of PPR motif in rice. (A) Typical structures of PPR proteins in rice. The number of motifs in each protein can vary from 2 to 28 in rice. (B) The consensus sequence of 10 PPR motif in rice. The overall height of each stack indicates the conservation of the sequence at that position and the bit score indicates the relative frequency of the corresponding acid. The lengths of the motifs can be estimated using the scale at the bottom. Figure S2. Distribution of PPR genes in rice chromosomes. The 491 PPR genes are widely and unevenly distributed on all 12 chromosomes of rice. Figure S3. Exon/intron structures of the PPR genes in rice. Yellow boxes represent exons, and black lines represent introns. Upstream or downstream regions are indicated by blue boxes. The sizes of exons and introns can be estimated using the scale at the bottom. Figure S4. Phylogenetic relationship of the PLS subgroup genes in rice and other species. Evolutionary relationships of PLS subgroup genes from rice, Arabidopsis, moss, tomato, and foxtail millet. The genes whose IDs start with LOC represent rice gene, AT represents Arabidopsis, Pp represents moss, Solyc represents tomato, and Seita represents foxtail millet. Figure S5. Expression patterns of PPR genes in different rice tissues. The FPKM expression values from RGAP of PPR genes at various developmental stages were log2 transformed, and a heat map was generated using the MeV4.9 software. Samples are indicated at the top of each lane: shoots, leaves-20 days, pre/post-emergence inflorescence (pre-EI, post-EI), anther, pistil, seed-5 DAP, seed-10 DAP, embryo- 25 DAP and endosperm- 25 DAP. The color scale (representing average log2 signal values) is shown at the top. (ZIP 6622 kb) [file 12864_2018_5088_MOESM2_ESM.zip › Figure S5.tiff]
